# Integrated analysis of transcriptomic and proteomic data from tree peony (*P. ostii*) seeds reveals key developmental stages and candidate genes related to oil biosynthesis and fatty acid metabolism

**DOI:** 10.1038/s41438-019-0194-7

**Published:** 2019-10-01

**Authors:** Xiaojing Wang, Haiying Liang, Dalong Guo, Lili Guo, Xiangguang Duan, Qishi Jia, Xiaogai Hou

**Affiliations:** 1 College of Agriculture / College of Tree Peony, Henan University of Science and Technology, Luoyang, 471023 China; 2 Department of Genetics and Biochemistry, Clemson University, Clemson, SC 29634-0318 USA; 3 College of Forestry, Henan University of Science and Technology, Luoyang, 471023 China

**Keywords:** Transcriptomics, Proteomics, Transcriptomics, Transcriptomics, Proteomics

## Abstract

Tree peony (*Paeonia* section *Moutan* DC.) seeds are an excellent source of beneficial natural compounds that promote health, and they contain high levels of alpha-linolenic acid (ALA). In recent years, tree peony has been emerging as an oil crop. Therefore, combined analysis of the transcriptome and proteome of tree peony (*P. ostii*) seeds at 25, 32, 39, 53, 67, 81, 88, 95, and 109 days after pollination (DAP) was conducted to better understand the transcriptional and translational regulation of seed development and oil biosynthesis. A total of 38,482 unigenes and 2841 proteins were identified. A total of 26,912 differentially expressed genes (DEGs) and 592 differentially expressed proteins (DEPs) were clustered into three groups corresponding to the rapid growth, seed inclusion enrichment and conversion, and late dehydration and mature stages of seed development. Fifteen lipid metabolism pathways were identified at both the transcriptome and proteome levels. Pathway enrichment analysis revealed that a period of rapid fatty acid biosynthesis occurred at 53–88 DAP. Furthermore, 211 genes and 35 proteins associated with the fatty acid metabolism pathway, 63 genes and 11 proteins associated with the biosynthesis of unsaturated fatty acids (UFAs), and 115 genes and 24 proteins associated with ALA metabolism were identified. Phylogenetic analysis revealed that 16 putative fatty acid desaturase (FAD)-encoding genes clustered into four *FAD* groups, eight of which exhibited the highest expression at 53 DAP, suggesting that they play an important role in ALA accumulation. RT-qPCR analysis indicated that the temporal expression patterns of oil biosynthesis genes were largely similar to the RNA-seq results. The expression patterns of fatty acid metabolism- and seed development-related proteins determined by MRM were also highly consistent with the results obtained in the proteomic analysis. Correlation analysis indicated significant differences in the number and abundance of DEGs and DEPs but a high level of consistency in expression patterns and metabolic pathways. The results of the present study represent the first combined transcriptomic and proteomic analysis of tree peony seeds and provide insight into tree peony seed development and oil accumulation.

## Introduction

Tree peony (*Paeonia* section *Moutan* DC.) is a perennial deciduous shrub that is well known for its large and colorful flowers with high ornamental, medicinal, and oil value. Previous studies on tree peony have mainly focused on its flowers, including the assessment of flower color^1,2^, cut flowers^3,4^, and genetic linkage mapping^5,6^, while little research has focused on its seeds. In recent years, tree peony, with oil-rich seeds, has received growing attention as an economically important oil crop. Depending on the species and variety, tree peony seeds contain 24.0–37.8% oil (w/w)^7,8^, and > 90% of their total fatty acids are unsaturated fatty acids (UFAs) such as oleic acid (OA), linoleic acid (LA) and alpha-linolenic acid (ALA). In particular, the proportion of ALA is quite high (~45%)^7^. ALA has been linked to a variety of health benefits, including reduced blood pressure, inhibition of platelet aggregation, and a lower risk of cancer^9,10^. The UFA content of tree peony seed oil complies with the international nutritional standards for “omega meals” and is similar to oil from olive and camellia trees, which are two of the world’s main oil-producing trees^11,12^. Tree peony seeds are also rich in polysaccharides, amino acids, and flavonoids^13,14^. Due to the potential value of tree peony seeds as a source of “healthy” oil, it is important to understand seed development and oil biosynthesis in peony to identify strategies for increasing oil content and quality through breeding and management practices.

Although considerable research has been conducted to improve the seed yield and UFA content^15,16^, there is limited information on the regulatory mechanisms controlling seed development and oil biosynthesis. RNA sequencing (RNA-seq) is a powerful tool for studying global gene expression and identifying genes that are functionally important under different physiological conditions or stages of development^17^. RNA-seq analysis has been used to examine the regulatory mechanisms associated with seed development, fatty acid biosynthesis, and UFA accumulation in woody oilseed crops such as olive, camellia, and *Jatropha curcas*^18–20^. However, such research has only recently been initiated in tree peony. The first comprehensive study characterizing gene expression in tree peony seeds was conducted by Li et al.^21^. They identified 388 unigenes that were potentially involved in fatty acid and triacylglycerol (TAG) biosynthesis. Yin et al.^22^ constructed six small-RNA libraries to identify noncoding RNAs (ncRNAs) involved in fatty acid synthesis and reported 318 known and 153 new miRNAs in tree peony seeds. However, these studies were limited to only one or a few stages of seed development. Oil accumulates in peony seeds as the seed develops, which takes approximately four months from pollination to seed maturity. Seed morphology and the level of chemical compounds vary greatly at different developmental stages, and the expression of genes and proteins related to fatty acid metabolism also varies^23^. Since gene expression is regulated at multiple levels, including posttranscriptional modification and differences in half-lives^24^, transcript abundance alone is not sufficient to infer the role of specific genes in cellular metabolism. Proteins are the principal mediators of metabolic activity and connect transcript expression to cellular metabolism. Therefore, combining transcriptome and proteome analyses is essential for developing a comprehensive understanding of the molecular mechanisms regulating seed development and fatty acid metabolism. However, integrated analyses based on both of these -omic approaches have rarely been reported for oil seed crops. Liu et al.^25^ used a proteomic approach to identify proteins associated with OA during peanut seed development. A time-series analysis of the transcriptome was used to characterize the molecular networks associated with oil accumulation in canola by Wan et al.^26^. That study identified key periods of fatty acid biosynthesis and numerous proteins associated with oil biosynthesis. Since the process of oil biosynthesis is very similar among oilseed plants^27^, the identification of these oil synthesis-related proteins also provides useful information for understanding the regulation of oil synthesis and fatty acid metabolism in tree peony seeds. However, tree peony seeds differ greatly from other oilseed plants in terms of their oil content and fatty acid composition^7^. Therefore, a specific study of the proteome of tree peony seeds is needed. An integrated analysis of transcriptomic and proteomic changes can provide a comprehensive overview of the dynamic processes regulating seed development and oil biosynthesis. Currently, no such integrated analysis has been conducted in tree peony seeds.

In the present study, a parallel analysis of the transcriptome and proteome of *P. ostii* seeds over their entire developmental time course was conducted using RNA sequencing and protein isobaric tagging (IBT) technology. Reverse transcription - quantitative PCR (RT-qPCR) and multiple-reaction monitoring (MRM) methods were also used to quantify relative gene expression and the abundance of proteins associated with fatty acid metabolism and seed development. The tree peony species *P. ostii* was selected because of its long history of cultivation and high seed yield and oil content. The main objective of the study was to augment the genomic and proteomic data available for tree peony and identify key stages of seed development and candidate genes involved in oil biosynthesis and fatty acid metabolism. The data obtained in this study provide a foundation for the development of strategies for improving seed yield and oil quality in tree peony.

## Materials and methods

### Plant material

**Fig. 1:**
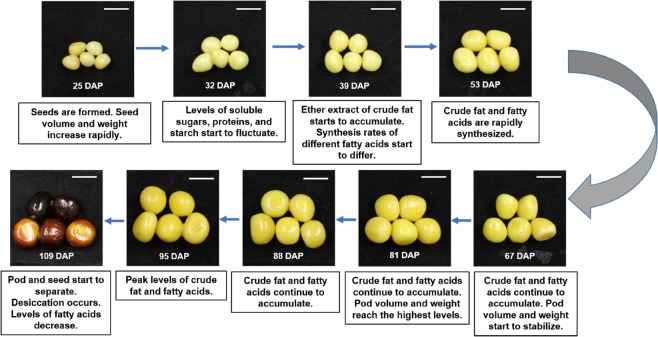
Nine developmental time points of *P*. *ostii* seeds included in the transcriptomic and proteomic studies. The characteristics of each time point were taken from Ma et al.^23^. The white barcode at the right top indicates 1 cm

The nine-year-old *P. ostii* plants used in the study were grown in a tree peony plantation located at Henan University of Science and Technology (112°36′19.65″E, 34°39′55.43″N), Luoyang, China. The seed development process from pollination through full maturation was observed from April to August 2016. Based on the parameters defined by Ma et al.^23^, seeds were collected at nine time points during their development (25, 32, 39, 53, 67, 81, 88, 95, and 109 DAP, collected on May 03, May 10, May 17, May 31, June 14, June 28, July 05, July 12 and July 26, 2016, respectively) (Fig. 1). Seeds collected from the same individuals were used for both RNA sequencing and proteome analyses. Pods at the same developmental stage were sampled from two different trees. The samples were frozen in liquid nitrogen immediately following collection and stored at −80 °C until further use.

### RNA extraction, cDNA library construction, high-throughput sequencing, raw data processing, and mapping

Total RNA was extracted from the seeds collected at the nine developmental time points using a TIANGEN RNAprep Pure Plant Kit (Tiangen Biotech Co. Ltd, Beijing, China) according to the manufacturer’s protocol and purified with a Dynabeads® Oligo (dT)25 kit (Life, USA) as described by Li et al.^21^. The concentration and quality of RNA were determined using an Agilent 2100 Bioanalyzer (Agilent Technologies, Santa Clara, CA, USA). Subsequently, mRNAs were obtained using Oligo (dT) magnetic beads and reverse transcribed into double-stranded cDNA (dscDNA) using an N6 random primer. Library quality was assessed with an Agilent Technologies 2100 Bioanalyzer prior to conducting single-end 50 bp read sequencing on the BGISEQ-500 platform. Low-quality reads containing >10% unknown bases or 50% low-quality bases (sequencing quality lower than 5) were removed from the dataset, and the remaining high-quality reads were mapped to tree peony reference genes available from the National Center for Biotechnology Information (NCBI) Transcriptome Shotgun Assembly database (BioSample accession no. SRS1180651, https://www.ncbi.nlm.nih.gov/biosample/?term = SRS1180651) with Bowtie2 (ref. ^28^).

### Quantification of gene expression, gene ontology, and KEGG pathway enrichment analysis

The relative levels of gene expression were analyzed with RSEM^29^ based on fragments per kilobase of transcript per million (FPKM) mapped read values. Correlation values were determined using the quantitative FPKM results, while the distances of expressed genes were calculated using the Euclidean method. Condition specificity analysis was performed using a statistical model previously described by Robinson and Oshlack^30^ and Yu et al.^31^. Enrichment of expression ≥ 5 and a *P*-value ≤ 0.001 were set as the default threshold to judge significant differences between time point-specific genes.

Differentially expressed genes (DEGs) were identified using the NOISeq method^32^ with the following default criteria: a fold change ≥ 2 and a divergence probability ≥ 0.8, with the May 03 sample (25 DAP) serving as the control. Hierarchical clustering was performed with the TIGR Multiple Experiment Viewer (MeV 4.9.0) (ref. ^33^).

All DEGs were mapped to GO terms (http://www.geneontology.org/) to conduct the gene ontology (GO) enrichment analysis. Gene numbers for each term were calculated, and a hypergeometric test^34^ was used to identify significantly enriched GO terms. The calculated *P*-value was adjusted via Bonferroni correction^35^, and a corrected *P*-value ≤ 0.05 was used as a threshold. The KEGG pathway-related database was used to perform pathway enrichment analysis of the DEGs^36^. A corrected *P*-value ≤ 0.05 was used as a threshold to define significantly enriched pathways associated with the DEGs.

### Expression patterns and phylogenetic analysis of *P. ostii* fatty acid desaturase (*FAD*) unigenes

The expression patterns of 16 annotated *P. ostii FAD* unigenes were displayed as a heat map by comparing FPKM reads. Hierarchical clustering of the unigenes and samples was conducted using a complete linkage approach. Translated *P. ostii* sequences with at least 90 amino acids were aligned to FADs from other species with ClustalW. Phylogenetic analysis was performed using the neighbor-joining (NJ) method. Bootstrap tests were performed with 1,000 replicates for statistical reliability. The phylogenetic analysis was carried out in MEGA 5.2 (ref. ^37^). FADs of *Arabidopsis thaliana*, *Brassica napus*, *Glycine max*, *Oryza sativa*, *Camellia sinensis*, *Paeonia lactiflora*, and *Paeonia suffruticosa* were obtained from Dong et al.^38^ and Yin et al.^39^.

### RT-qPCR analysis of genes involved in oil biosynthesis

Total RNA was isolated from seeds collected at nine different developmental time points as previously described. Then, using 1 μg of total RNA, cDNA was prepared for RT-qPCR using All-in-one First-Strand cDNA Synthesis SuperMix (TransGen, Beijing, China) according to the manufacturer’s instructions. Genomic DNA was removed with the DNase and gDNA Remover included in the commercial kit. PCR primers were designed with Oligo 7.0 software for sequences encoding fatty acid desaturase genes (*SAD*, *FAD2*, *FAD3*, *FAD6*, *FAD7*, *FAD8*) and phospholipids:diacylglycerol acyltransferase genes (*PDAT1*, *PDAT2*). All RT-qPCR assays were performed using Top Green qPCR SuperMix (TransGen, China) in a LightCycler 480II Real-Time PCR System (Roche, Mannheim, Germany). The cycling conditions were 95 °C for 15 min, followed by 35 cycles of 95 °C for 10 s and 60 °C for 20 s. Six replicates were analyzed for each target gene. The ubiquitin gene was used as an internal control^40^. The relative expression levels of target genes were calculated using the 2^-ΔΔCt^ comparative threshold cycle (Ct) method^41^.

### IBT-based quantitative proteomics of developing tree peony seeds

Total proteins from tree peony seeds collected at nine different developmental time points were extracted according to a previously described protocol^42^. Briefly, trypsin Gold (Promega, Madison, WI, USA) was used to digest the proteins overnight at 37 °C with a 40:1 ratio of protein:trypsin. This was followed by desalination with a Strata X C18 column (Phenomenex). IBT labeling reagents (2 mg)^43^ were dissolved in 80 μL of isopropanol before being combined with samples.

The labeled peptides were separated using a Shimadzu LC-20AB HPLC Pump system coupled with a high pH RP column as described by Sun et al.^44^. The eluted peptides were monitored by measuring the absorbance at 214 nm and pooled into 20 fractions before being loaded onto a C18 trap column using a LC-20AD nano-HPLC instrument (Shimadzu, Kyoto, Japan). The separated peptides were then analyzed by tandem mass spectrometry (MS) on a Q EXACTIVE mass spectrometer (Thermo Fisher Scientific, San Jose, CA) for data-dependent acquisition detection by nanoelectrospray ionization. The parameters for MS analysis were the same as those listed in Sun et al.^44^.

After format conversion, the MS/MS data were searched against the tree peony reference database indicated above using Mascot (version 2.3.02) (ref. ^45^). iQuant^46^ was used for quantitatively analyzing the isobaric tag-labeled peptides. A false discovery rate (FDR) of 1% and the picked protein FDR strategy^47^ were employed.

### GO, pathway enrichment, and clustering analyses of differentially expressed proteins (DEPs)

A DEP was defined as being significantly regulated if the *Q*-value was less than 0.05 and the fold change was at least 1.5. GO, pathway enrichment, and clustering analyses of the DEPs were conducted as previously described for DEGs.

### Relative quantification of target proteins via multiple-reaction monitoring (MRM)

Seven proteins determined to be differentially expressed by both gene expression and proteomic analyses were selected for qualitative and quantitative analysis via MRM to validate the IBT results. The selected proteins were 3-oxoacyl-[acyl-carrier-protein] synthase III (*KAS3A*, Unigene844_All), fatty acyl-ACP thioesterase A (*FATA*, Unigene25499_All), NAD-dependent aldehyde dehydrogenase family 2 member B7 (*ALDH2B7*, Unigene12082_All), Acyl-CoA-binding protein (*ACBP*, Unigene28921_All), seed maturation protein PM34 (*PM34*, Unigene7230_All), oleosin (Unigene6102_All), and the GDSL esterase/lipase protein (*GELP*, Unigene29949_All). The ginseng β-galactosidase protein (sp|P00722|BGAL_ECOLI) was utilized for data normalization, minimizing the experimental error of MRM nonstandard quantification^48,49^.

Seed samples representing different developmental stages were homogenized for protein extraction as described above. The extracted protein (100 μg) was digested at 37 °C for 16 h with Trypsin Gold using a 30:1 ratio of protein:trypsin. Each sample was spiked with 50 fmol of β-galactosidase for normalization. DTT (10 mM) was added to reduce disulfide bonds in proteins. MRM analysis was performed on a QTRAP 5500 LC-MS/MS system (AB SCIEX, Framingham, MA, USA) equipped with an LC-20AD nanoHPLC system (Shimadzu, Kyoto, Japan). The chromatography and mass spectrometry conditions were the same as those described by Li et al.^50^. Multiple MRM transitions were monitored using unit resolution at both the Q1 and Q3 quadrupoles to maximize specificity.

Skyline software was used to integrate the raw file generated by the QTRAP 5500 system. Indexed retention time (iRT) normalization was used to identify the chromatograph of a given peptide against a spectral library. All transitions for each peptide were used for quantitation unless interference from the matrix was observed. MSstats with a linear mixed-effects model were employed. *P*-values were adjusted to control the FDR at a cutoff of 0.05. All proteins with a *P*-value below 0.05 and a fold change larger than 1.5 were considered significant.

## Results

### Transcriptome analysis

#### RNA sequencing, alignment of expressed unigenes, and developmental stage-specific genes

Eighteen libraries from seeds collected at nine stages of development were sequenced to provide a profile of the global transcriptome of *P. ostii* tree peony seeds. A total of 21.7 billion base pairs were obtained, with an average of 24,137,232 raw sequencing reads and 24,093,307 clean reads per sample after the removal of poly-N sequences and low-quality reads. The clean reads are available in the NCBI Short Read Archive (accession number: PRJNA495085). The average ratio of clean reads to raw reads was 99.77%. The clean reads were mapped to tree peony reference genes, with an average mapping percentage of 53.70% per sample. Overall, 37.45–45.39% of the reads mapped to a single location, while 9.01–18.03% mapped to multiple positions (Supplementary Table S1). A total of 38,482 unigenes were identified. The proportion of unigenes in each sample ranged from 66% to 73.72%, with the percentage exhibiting a downward trend as seeds developed and matured (Supplementary Fig. S1).

**Fig. 2:**
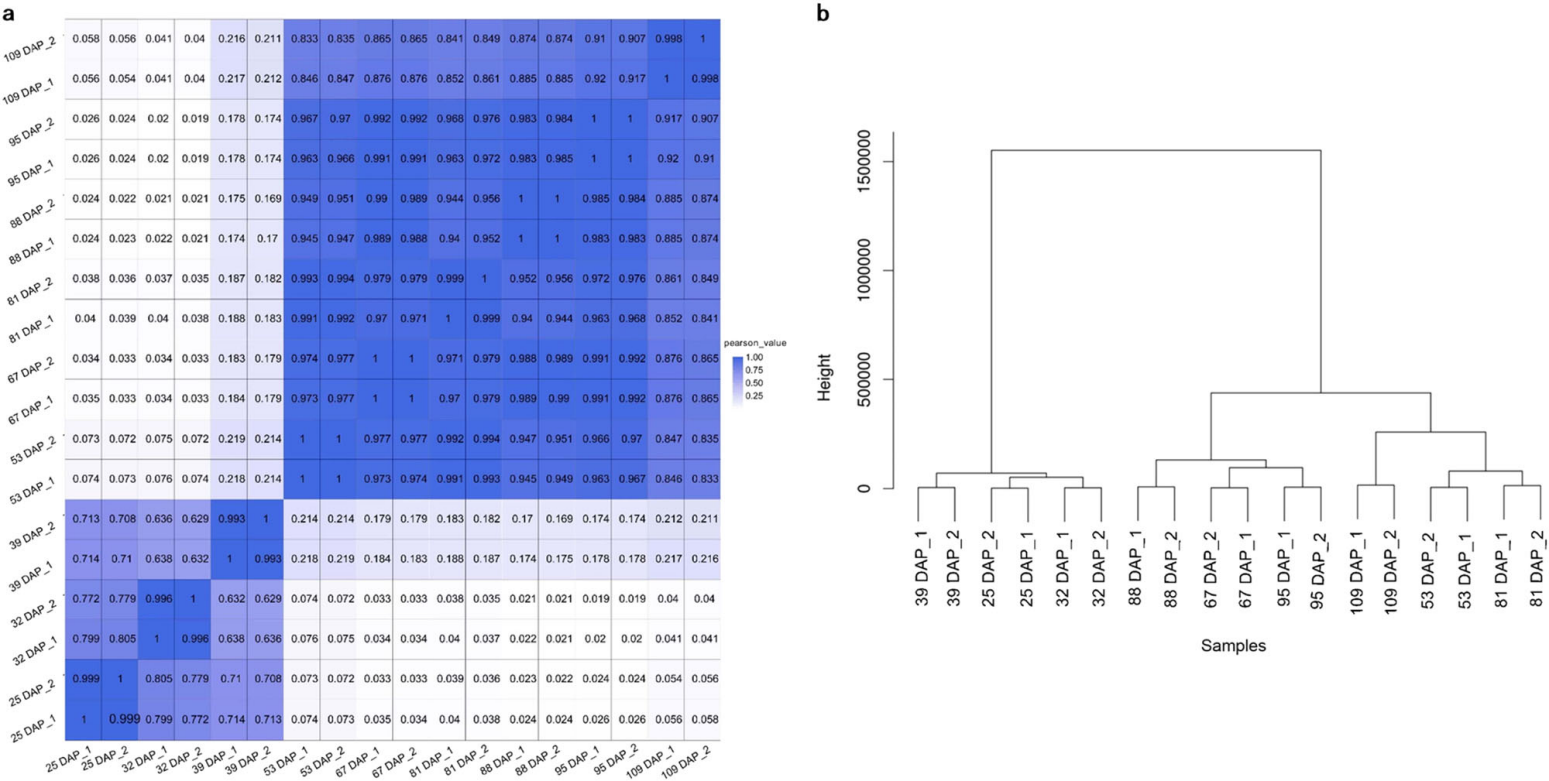
Correlation of RNA-seq data among 18 *P. ostii* seed samples. **a** Heat map of correlation coefficient values across samples based on RNA-seq FPKM. **b** Cluster tree based on the distances of expressed genes

Samples of seeds were obtained from two peony trees at each time point, and each tree served as a biological replicate. The correlation value calculated from the FPKM values of each sample revealed that the two samples collected at each time point exhibited a correlation value of at least 0.993, indicating that there was a high level of uniformity between the biological replicates (Fig. 2a). A cluster tree based on the distances between expressed genes is presented in Fig. 2b. The total samples were divided into two main groups: samples from the first three time points (25, 32, and 39 DAP) constituted one group, and the remaining samples formed the second group. The separation of the two groups was also evident in the correlation heat map (Fig. 2a). The correlation values between these two groups of samples were very low, ranging from 0.019 to 0.219, indicating that a broad differences in gene expression existed between these two groups of samples. The identified grouping was consistent with the timeline and stages of the early seed development vs. maturation of tree peony seeds^23^.

Condition-specific expression analysis was conducted to identify genes that were uniquely expressed at each time point of seed development. The 109 DAP sample, representing mature seeds, exhibited the greatest number of unique genes, 271 and 278 in tree 1 and tree 2, respectively, which highlights the distinction between the transcriptome of mature seeds and the transcriptome of developing seeds. The number of unique genes in the seeds at 25, 39, 81, 88, and 95 DAP ranged from 22 to 49, and that in the seeds at 32, 53, and 67 DAP ranged from 3 to 11. The unique genes that were common to the seeds of both peony trees are listed in Supplementary Table S2. It is important to note that metabolic pathways and the biosynthesis of secondary metabolites were enriched among the 215 common unique genes identified in the 109 DAP mature seeds. Functional analysis of these unique genes can help reveal the specific biological processes occurring at a particular stage of seed development and identify RNA biomarkers.

#### Differential expression analysis and clustering of DEGs

**Fig. 3:**
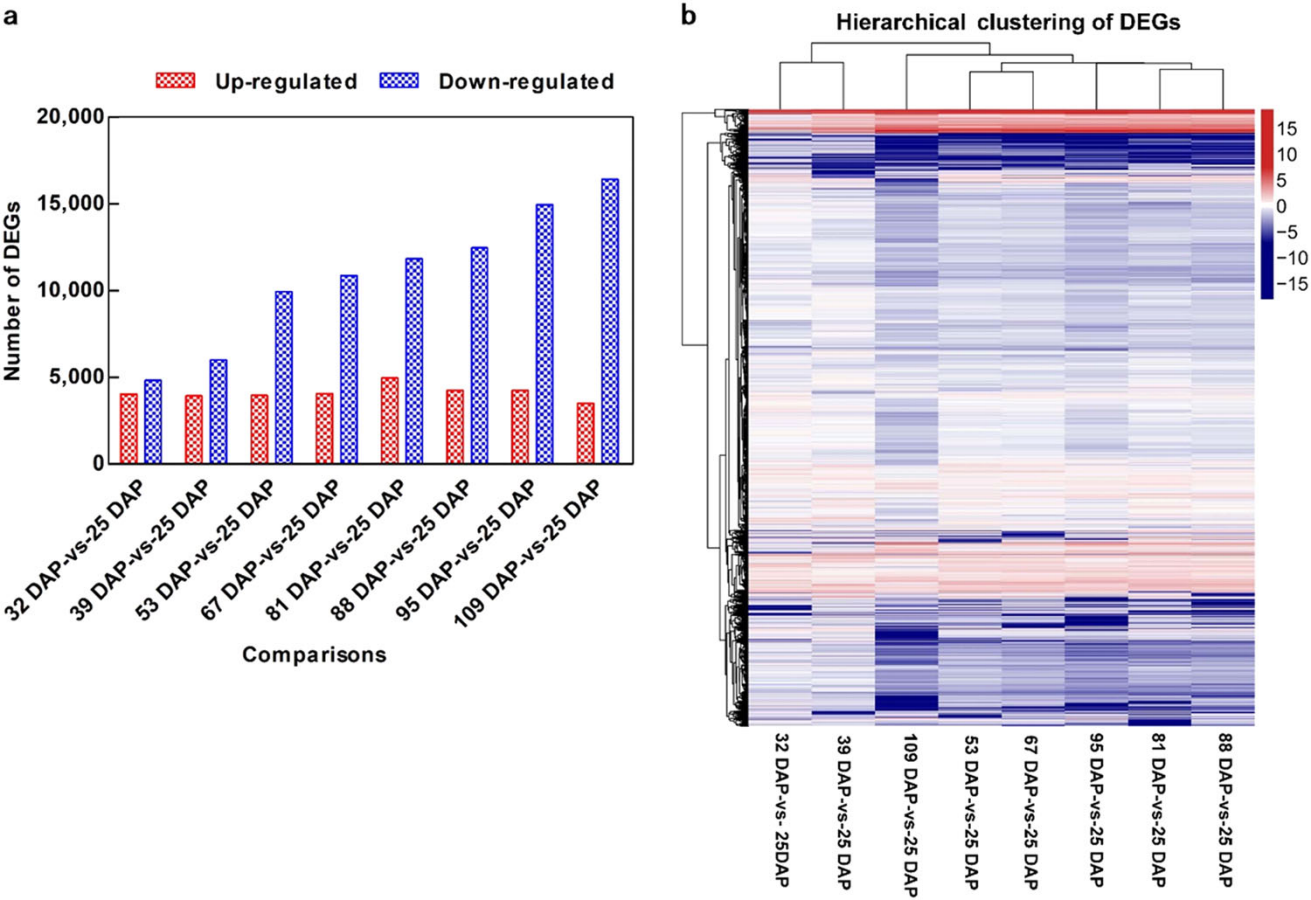
Differentially expressed genes (DEGs) identified in *P*. *ostii* seeds of various development stages. **a** Quantity of DEGs. **b** Cluster analysis of DEGs. The gradient-colored barcode at the top right indicates the log_2_(FC) value. FC, Fold change in expression at a particular time point relative to expression in 25 DAP seeds

Differential expression analysis was conducted to characterize the temporal changes in global gene expression. A total of 4017, 3924, 3955, 4055, 4966, 4236, 4242, and 3484 upregulated genes and 4831, 6016, 9926, 10,861, 11,820, 12,457, 14,941, and 16,403 downregulated genes were identified when 25 DAP seeds were compared to seeds from successive later time points (Fig. 3a). The number of upregulated genes was similar at the different time points; however, more downregulated genes were observed as the seeds matured. The eight comparisons with the 25 DAP sample could be grouped into three main clusters based on the patterns of differential gene expression: 32–39 DAP, 53–95 DAP, and 109 DAP (Fig. 3b). The clustered time points suggest that the tree peony seeds were at a similar developmental stage at the time points within each of the clusters.

#### GO functional classification and enrichment analysis of DEGs

GO functional annotation was carried out on the DEGs identified in the eight comparisons with the 25 DAP sample (Supplementary Fig. S2). The eight comparisons produced similar representations of GO functional categories. The GO annotations of the DEGs were classified into 42 functional groups within the categories of biological processes, cellular components, and molecular functions. Among the 20 groups within the biological process category, most DEGs were cataloged under metabolic processes and cellular processes, followed by single-organism processes and localization. Within the 12 groups in the cellular components category, the cell and cell part categories were relatively dominant, followed by organelle and membrane groups. The catalytic activity and binding groups were the most highly represented in the ten groups within the molecular functions category.

While the eight comparisons exhibited similar patterns of GO functional classification, they differed in their enrichment classification (Supplementary Table S3). Enriched biological processes were found in five comparisons, and four of these comparisons included an enriched process involved in the organization of DNA and protein-related structures such as nucleosome and protein-DNA complex subunit organization, underscoring the significance of gene regulation during seed development. The phenylpropanoid biosynthetic process and secondary metabolite biosynthetic process categories were enriched in the 53 DAP-vs-25 DAP comparison (when crude fat and various fatty acids were rapidly being synthesized. Metabolic processes were enriched in the 95 DAP samples (when the levels of crude fat and various fatty acids peaked). Enriched GO cellular components were found in all eight comparisons, including chromatin, plastids, membranes, endoplasmic reticulum, mitochondrion, ribonucleoprotein complexes, and macromolecular complexes. Notably, in the 109 DAP samples (when desiccation occurs and the levels of fatty acids start to decrease), degradation complexes (proteasome, peptidase, and endopeptidase complexes) were enriched. Five of the eight comparisons produced enriched GO molecular functions. Structural molecules and hydrolase activity were abundant at the sampling times when crude fat and various fatty acids accumulated and peaked. Enrichment of catalytic and oxidoreductase activity was evident when crude fat and fatty acids started to decrease. Hydrolase, carboxypeptidase, iron ion binding, oxidoreductase, and glucosidase activity were enriched when crude fat started to accumulate, and the rates of the synthesis of different fatty acids began to vary (39 DAP). Overall, the results of the GO enrichment analysis of DEGs corroborate the accumulation of sugars, proteins, and fatty acids in developing *P. ostii* seeds reported by Li et al.^21^ and Ma et al.^23^.

#### KEGG functional classification and pathway enrichment analysis of DEGs

KEGG pathway analysis was conducted to identify the major active biological pathways in developing tree peony seeds. The DEGs were assigned to 21 KEGG terms under the following six primary categories: cellular processes, environmental information processing, genetic information processing, human diseases, metabolism, and organismal systems (Supplementary Fig. S3). The global and overview maps contained the largest number of DEGs in all eight comparisons, followed by carbohydrate metabolism, with the exception of the 95 DAP-vs-25 DAP comparison, in which translation exhibited the second highest number of DEGs. Overall, metabolism and genetic information processing presented the most DEGs in the eight comparisons, ranging from 62% to 65% and 17% to 23%, respectively.

Enrichment analysis was performed on the 134 pathways to which the DEGs were mapped to elucidate the enriched biological function of each comparison (Table 1). Photosynthesis, DNA replication, and ribosome pathways were enriched in the 32–67 DAP samples, when seeds were rapidly developing and before pod volume and weight peaked. The flavonoid biosynthesis pathway was significantly enriched in all comparisons of seeds performed prior to the peak accumulation of crude fat and fatty acids (95 DAP). The starch and sucrose metabolism pathways were enriched in the 39 DAP and 53 DAP samples when crude fat and fatty acids began to accumulate and were rapidly synthesized. Fatty acid biosynthesis was also found to be enriched in the 53 DAP samples. The only enriched pathway identified after the accumulation of crude fat and fatty acids peaked (95 DAP) was the ribosome pathway. Thereafter, proteasome and carbon metabolism and posttranslational modification pathways (N-glycan biosynthesis, glycosylphosphatidylinositol (GPI)-anchor biosynthesis) were enriched. Similar to the GO enrichment results, the KEGG enrichment analysis of DEGs corroborated the results regarding the levels of nutrients in developing tree peony seeds of *P. ostii* reported by Li et al.^21^ and Ma et al.^23^.

**Table 1:** Significantly enriched KEGG pathways of DEGs in *P. ostii* seeds from different development periods

Pathway	Pathway
**32 DAP-vs- 25 DAP**	**81 DAP-vs- 25 DAP**
Photosynthesis –antenna proteins	Ribosome
Photosynthesis	Flavonoid biosynthesis
DNA replication	Other glycan degradation
Glycosaminoglycan degradation	
Ribosome	
Flavonoid biosynthesis	
**39 DAP-vs- 25 DAP**	**88 DAP -vs- 25 DAP**
Starch and sucrose metabolism	Ribosome
Plant hormone signal transduction	Flavonoid biosynthesis
Photosynthesis –antenna proteins	
Flavonoid biosynthesis	
Phenylpropanoid biosynthesis	
Brassinosteroid biosynthesis	
Carbon fixation in photosynthetic organisms	
**53 DAP-vs- 25 DAP**	**95 DAP-vs- 25 DAP**
Flavonoid biosynthesis	Ribosome
Photosynthesis –antenna proteins	
Starch and sucrose metabolism	
Plant hormone signal transduction Fatty acid biosynthesis	
**67 DAP-vs- 25 DAP**	**109 DAP-vs- 25 DAP**
Ribosome	N-Glycan biosynthesis
DNA replication Flavonoid biosynthesis	Glycosylphosphatidylinositol(GPI)-anchor biosynthesis
Photosynthesis –antenna proteins	Proteasome Carbon metabolism

**Fig. 4:**
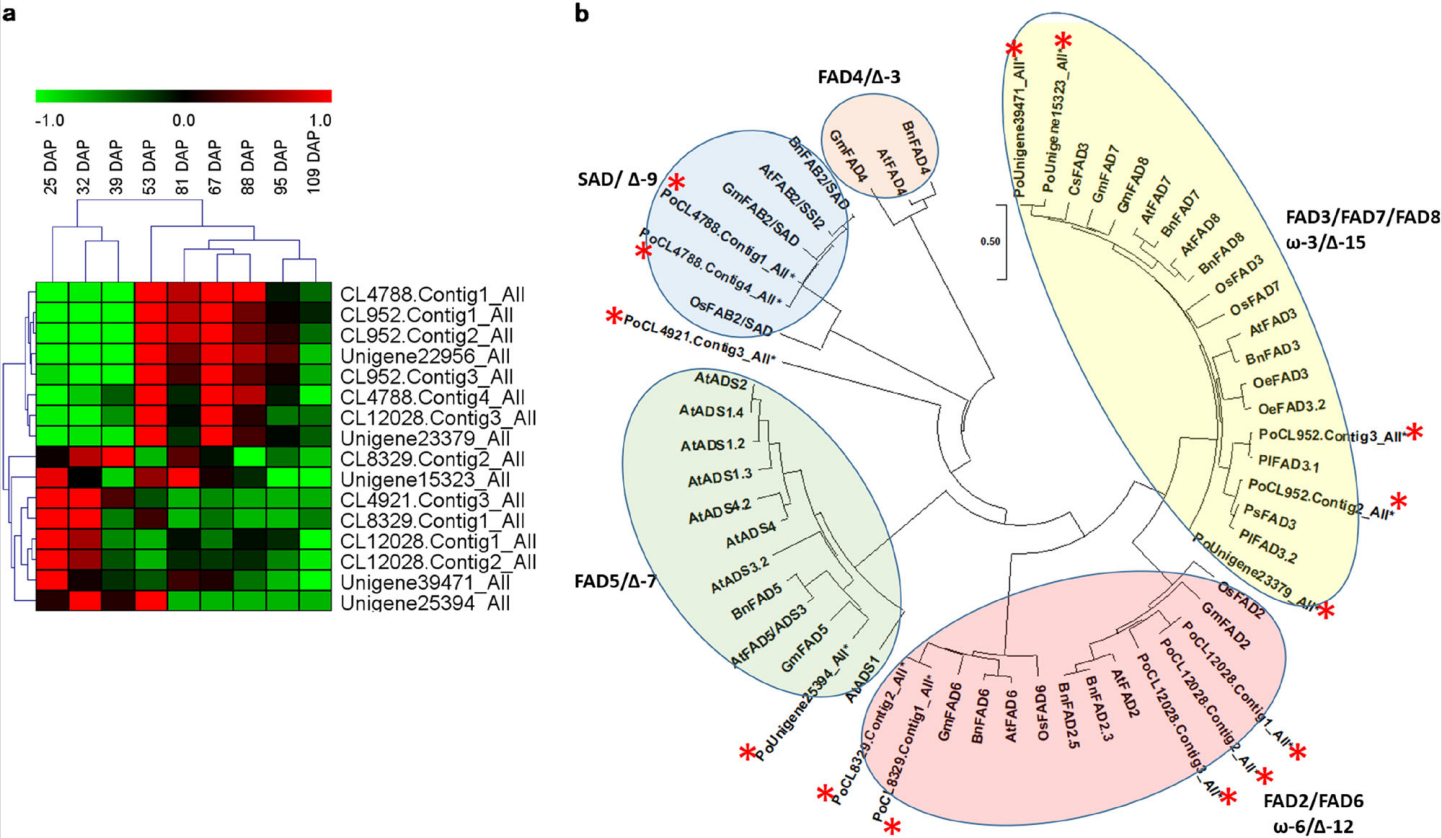
Putative fatty acid desaturase (*FAD*) unigenes identified in *P*. *ostii* seeds. **a** Heat map showing relative expression levels. The color scale (−1.0 to 1.0) represents the calculated Z-score. The hierarchical clustering of unigenes and samples is shown in the dendrogram on the top and side of the heat map using the complete linkage approach. **b** Phylogenetic tree depicting the relationship of *P. ostii FADs* with homologs from other species. At: *Arabidopsis thaliana*, Bn: *Brassica napus*, Gm: *Glycine max*, Os: *Oryza sativa*, Cs: *Camellia sinensis*, Pl: *Paeonia lactiflora*, Ps: *Paeonia suffruticosa*, Po: *Paeonia ostia*. The protein sequences were aligned using ClustalW, and the neighbor-joining (NJ) tree was constructed with the program MEGA 5. The *P. ostii FAD* proteins are indicated with asterisks. The five groups of *FAD* proteins are designated according to Dong et al.^38^ and Yin et al.^39^

Notably, the unigenes identified in the present study mapped to 15 of the 17 pathways for lipid metabolism (Supplementary Table S4). More specifically, 211 unigenes were associated with fatty acid metabolism, 93 with fatty acid biosynthesis, 131 with fatty acid elongation, 98 with fatty acid degradation, 63 with UFA biosynthesis, 46 with linoleic acid metabolism, 115 with ALA metabolism, 306 with glycerophospholipid metabolism, and 306 with glycerolipid metabolism. All of the 16 annotated *FADs* were differentially expressed at a minimum of one time point. An expression heat map revealed that these *FADs* could be grouped into two categories, with the first category exhibiting an increase in expression at the beginning of seed development (25–32 DAP) and the second exhibiting an increase in expression at later stages of seed development (53–95 DAP) (Fig. 4a). A phylogenetic analysis indicated that at least one *P. ostii* homolog is present in four of the five *FAD* groups that have been characterized in *Arabidopsis* (Fig. 4b). Interestingly, the CL4921. Contig3_All unigene formed a separate clade by itself. This unigene was only expressed at the first three time points of seed development. The identification of DEGs linked to lipid metabolism at different time points of tree peony seed development can provide important information about key stages of oil accumulation and the mechanism regulating UFA synthesis.

#### RT-qPCR analysis of oil biosynthesis genes during the nine time points of *P. ostii* seed development

**Fig. 5:**
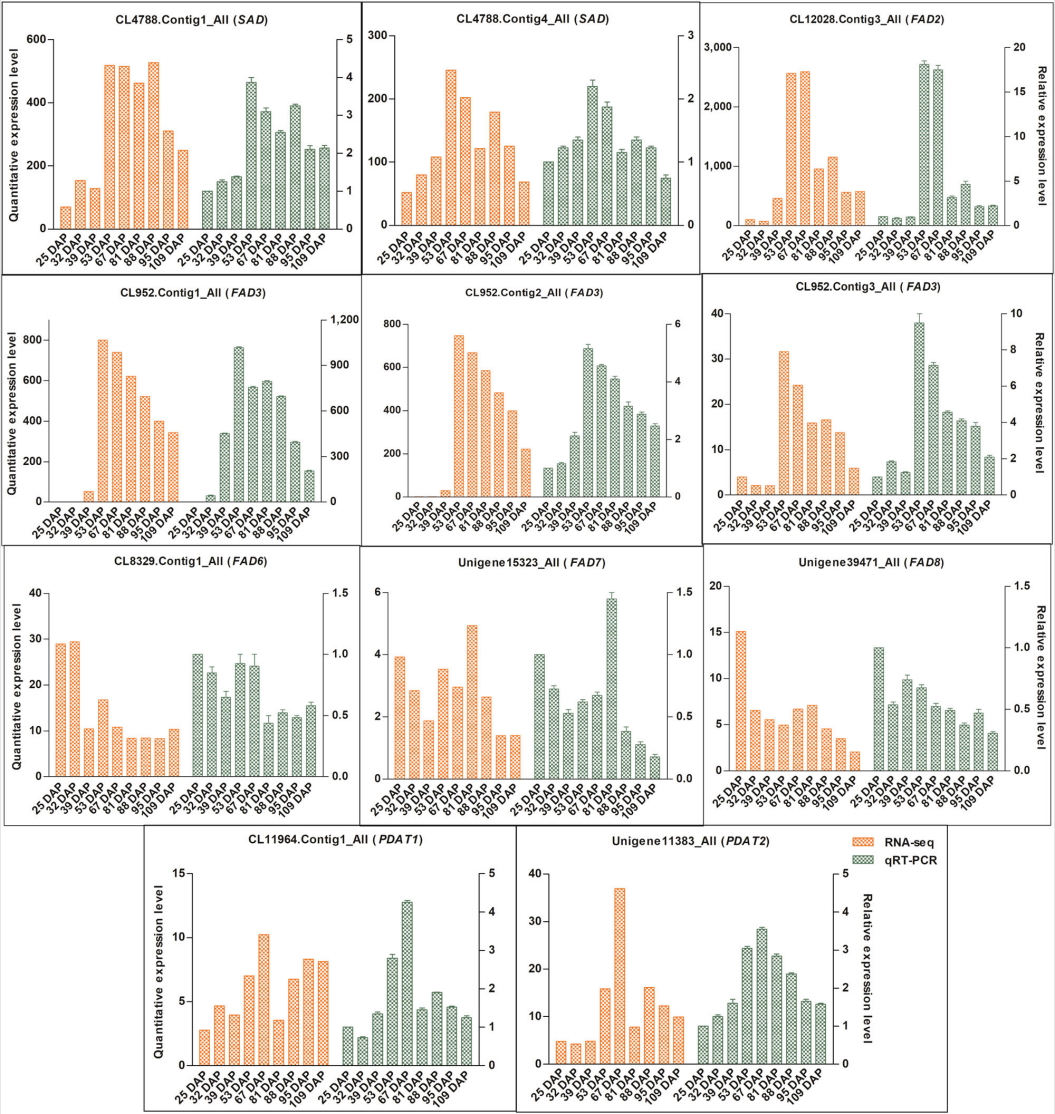
Relative expression levels of oil biosynthesis genes at nine time points of *P*. *ostii* seed development detected by RNA-seq and qRT-PCR

Nine *FAD* genes and two *PDAT* genes were selected for RT-qPCR analysis, including two *SAD* genes, three *FAD3* genes, *FAD2*, *FAD6*, *FAD7*, *FAD8*, *PDAT1*, and *PDAT2* (Fig. 5). *PDAT* is one of the key enzymes involved in triglyceride synthesis and catalyzes the synthesis of lysophospholipids and triacylglycerol from phospholipids and diacylglycerol. The results of the RT-qPCR analysis indicated that *SAD*, *FAD2*, *FAD3*, *PDAT1* and *PDAT2* were highly expressed from 53 DAP to 67 DAP, while *FAD6* and *FAD8* were highly expressed from 25 DAP to 32 DAP, and *FAD7* reached its peak expression at 81 DAP. Overall, the expression of oil biosynthesis genes as determined by RT-qPCR was similar to the RNA-seq results, except for small differences in expression values at some time points. These results confirm the reliability of the RNA-seq data obtained in the present study.

### Proteome analysis

#### Functional classification of proteins via GO and KEGG enrichment analyses

The proteomes of *P. ostii* seeds were characterized at the same nine time points that were examined to characterize gene expression. Collectively, 7026 peptides and 2841 proteins were identified with a 1% FDR (Supplementary Table S5). A total of 1995 proteins could be assigned GO annotations, which were classified into 46 groups within the three major GO categories (biological processes, cellular components, and molecular functions) (Supplementary Fig. S4). Among the 22 groups within the biological process category, the metabolic process and cellular process terms were associated with the most proteins, 1347 and 1157, respectively, followed by single-organism process (500) and response to stimulus (384). Among the 11 groups within cellular components, the cell (1071) and cell part (1071) terms were relatively dominant, followed by organelle (719) and membrane (571). Among the 13 groups within the molecular function category, catalytic activity (1307) and binding (999) were the most represented. All of the most highly represented terms identified were largely consistent with the results obtained in the analysis of gene expression.

The results of the KEGG pathway analysis indicated that 2475 proteins could be assigned to 132 pathways (Supplementary Table S6). The three pathways with the most proteins were metabolic pathways (821), biosynthesis of secondary metabolites (483), and carbon metabolism (174).

#### Quantitative statistics and cluster analysis of DEPs

**Fig. 6:**
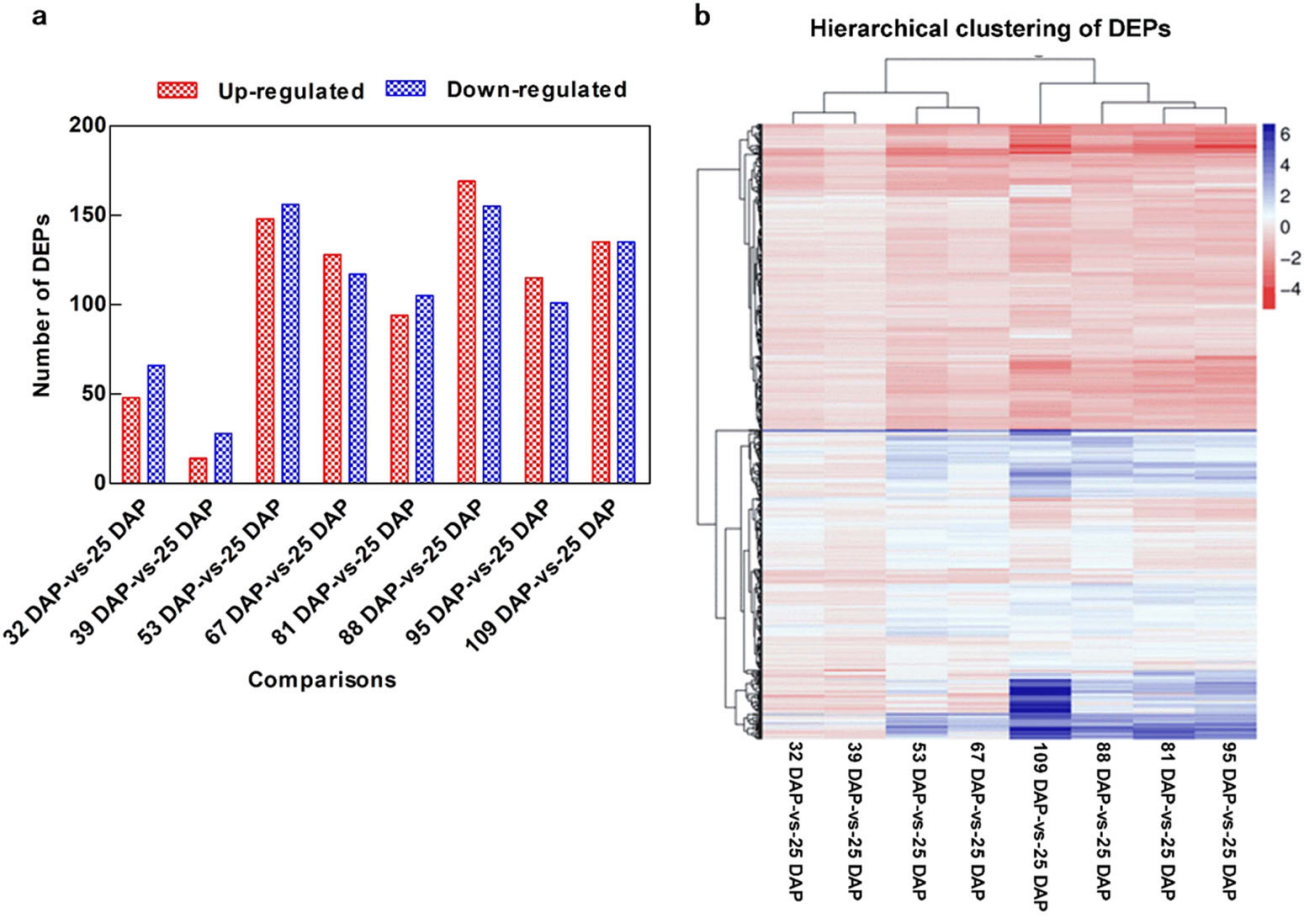
Differentially expressed proteins (DEPs) identified in *P*. *ostii* seeds from various development stages. **a** Numbers of DEPs. **b** Cluster analysis of DEPs. The gradient-colored barcode at the top right indicates the log_2_(FC) values. FC, Fold change in expression in the treatment group relative to expression in the control group

Relative to the 25 DAP time point, there were 48, 14, 148, 128, 94, 169, 115, and 135 upregulated DEPs detected in seeds collected at the eight subsequent time points, respectively. Additionally, 66, 28, 156, 117, 105, 155, 101, and 135 downregulated DEPs relative to seeds collected at 25 DAP were identified in the eight subsequently time points, respectively (Fig. 6a). Similar to the DEG heat map, the DEP heat map contained three major clusters based on the stages of seed development: 32–67 DAP formed one cluster; 81–95 DAP formed the second cluster; and 109 DAP formed the third cluster (Fig. 6b).

#### KEGG pathway enrichment analysis of DEPs

Enrichment analysis of KEGG pathways was performed to reveal enriched biological functions. When the eight successive time points (32, 39, 53, 67, 81, 88, 95, 109 DAP) were compared to the 25 DAP sample, 9, 3, 22, 26, 5, 18, 8, and 19 significantly enriched pathways, respectively, were identified in the collected samples (Table 2). These results were consistent with the pathway enrichment analysis of the DEGs. The results indicated that the flavonoid biosynthesis and ribosome pathways started to become enriched at the protein level at 32 DAP. DEP fatty acid biosynthesis pathways were enriched at 53, 67, 88, and 109 DAP. Six out of the eight comparisons revealed enriched KEGG pathways that were common to the DEGs and DEPs, indicating a high correlation between these two datasets. The discrepancies that were observed between the DEPs and DEGs were most likely due to the differences in timing between transcription and translation as well as posttranslational modifications. For example, starch and sucrose metabolism started to become enriched at 39 DAP in the DEGs and at 53 DAP in the DEPs. DEG enrichment of brassinosteroid biosynthesis was only evident at 39 DAP; however, enrichment of DEPs related to brassinosteroid biosynthesis was evident in all of the first four time point comparisons (32–67 DAP). DEP enrichment of the ALA and linoleic acid metabolism pathways was evident in four comparisons (32 DAP-, 67 DAP-, 88 DAP-, and 109 DAP-vs-25 DAP). Notably, enrichment of the mitogen-activated protein kinase (MAPK) signaling pathway was observed at the last four time points, substantiating its role in seed development.

**Table 2:** Significantly enriched KEGG pathways of the DEPs of *P. ostii* seeds from different development periods

Pathway	Pathway
**32 DAP-vs- 25 DAP**	**81 DAP-vs- 25 DAP**
Terpenoid backbone biosynthesis	Protein processing in endoplasmic reticulum
Brassinosteroid biosynthesis	Phagosome
Thiamine metabolism	Amino sugar and nucleotide sugar metabolism
Nitrogen metabolism	Tryptophan metabolism
Flavonoid biosynthesis	
ALA metabolism	
Fatty acid degradation	
Ribosome	**88 DAP-vs- 25 DAP**
Tryptophan metabolism	Metabolic pathways
**39 DAP-vs- 25 DAP**	Glycolysis/Gluconeogenesis
Ribosome	Protein processing in endoplasmic reticulum
Tryptophan metabolism	Carbon metabolism
Brassinosteroid biosynthesis	Fatty acid biosynthesis
**53 DAP-vs- 25 DAP**	Carbon fixation in photosynthetic organisms
Metabolic pathways	Fatty acid metabolism
Fatty acid biosynthesis	Starch and sucrose metabolism
Carbon metabolism	Pyruvate metabolism
Fatty acid metabolism	Fatty acid degradation
Carbon fixation in photosynthetic organisms	Flavonoid biosynthesis
Protein processing in endoplasmic reticulum	Biosynthesis of secondary metabolites
Starch and sucrose metabolism	Amino sugar and nucleotide sugar metabolism
Biosynthesis of secondary metabolites	Biotin metabolism
Flavonoid biosynthesis	Linoleic acid metabolism
Tyrosine metabolism	Peroxisome
Amino sugar and nucleotide sugar metabolism	Phagosome
Peroxisome	MAPK signaling pathway - plant
Fatty acid degradation	
Thiamine metabolism	
Glycolysis / Gluconeogenesis	
Isoquinoline alkaloid biosynthesis	**95 DAP-vs- 25 DAP**
Arachidonic acid metabolism	Protein processing in endoplasmic reticulum
Riboflavin metabolism	Phenylpropanoid biosynthesis
Brassinosteroid biosynthesis	Phagosome
Pyruvate metabolism	RNA polymerase
Tropane, piperidine and pyridine alkaloid biosynthesis	MAPK signaling pathway-plant
Phenylalanine, tyrosine and tryptophan biosynthesis	Metabolic pathways
**67 DAP-vs- 25 DAP**	Starch and sucrose metabolism
Metabolism pathways	Sphingolipid metabolism
Carbon fixation in photosynthetic organisms	
Fatty acid biosynthesis	**109 DAP-vs- 25 DAP**
Glycolysis / Gluconeogenesis	Protein processing in endoplasmic reticulum
Fatty acid metabolism	Metabolic pathways
Biosynthesis of secondary metabolites	Phagosome
Carbon metabolism	ALA metabolism
ALA metabolism	Biotin metabolism
Fatty acid degradation	Biosynthesis of secondary metabolites
Terpenoid backbone biosynthesis	Glycolysis/Gluconeogenesis
Cutin, suberine and wax biosynthesis	Flavonoid biosynthesis
Tyrosine metabolism	MAPK signaling pathway - plant
Thiamine metabolism	Starch and sucrose metabolism
Flavonoid biosynthesis	Fatty acid degradation
Protein processing in endoplasmic reticulum	Ascorbate and aldarate metabolism
Linoleic acid metabolism	Amino sugar and nucleotide sugar metabolism
Isoquinoline alkaloid biosynthesis	Peroxisome
Peroxisome	
Arachidonic acid metabolism	
Pyruvate metabolism	
Brassinosteroid biosynthesis	
Tropane, piperidine and pyridine alkaloid biosynthesis	
Tryptophan metabolism	
Biosynthesis of amino acids	
Biotin metabolism	
Phenylalanine metabolism	

Fifteen lipid metabolism pathways, identical to those identified in the transcriptome, were also identified in the proteome (Supplementary Table S4). However, the number of identified proteins was relatively small relative to the number of DEGs and included 35 proteins associated with fatty acid metabolism, 26 with fatty acid biosynthesis, 7 with fatty acid elongation, 31 with fatty acid degradation, 11 with UFA biosynthesis, 11 with linoleic acid metabolism, 24 with ALA metabolism, 27 with glycerophospholipid metabolism, and 24 with glycerolipid metabolism. The functions of these proteins need to be validated in future studies.

#### Targeted profiling of fatty acid metabolism- and seed development- related proteins

**Fig. 7:**
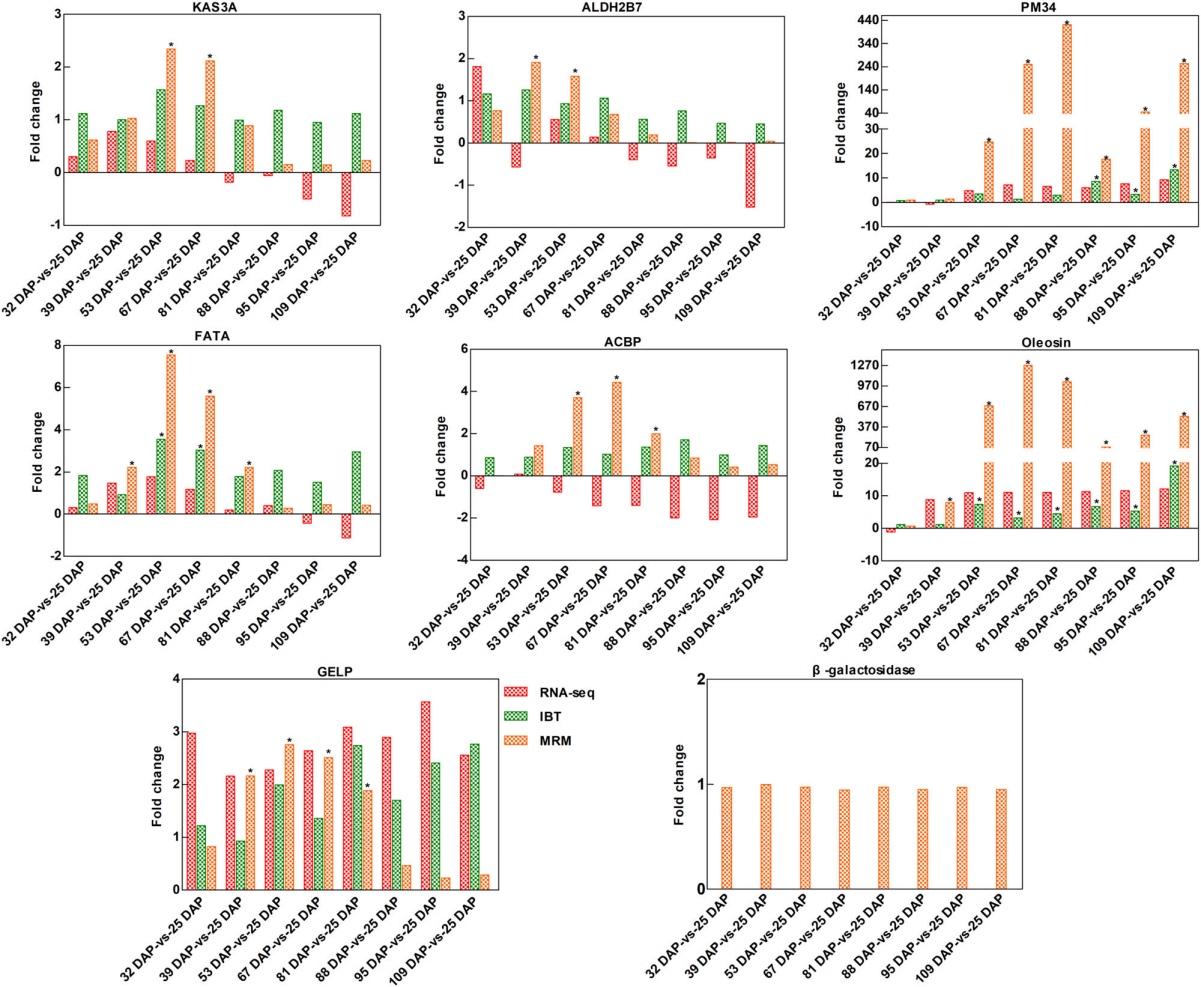
Relative quantification of seven proteins at different stages of seed development by MRM. The ginseng β-galactosidase protein (sp|P00722|BGAL_ECOLI) was utilized for data normalization. KAS3A: 3-oxoacyl-[acyl-carrier-protein] synthase III, FATA: fatty acyl-ACP thioesterase A, ALDH2B7: NAD-dependent aldehyde dehydrogenase family 2 member B7, ACBP: Acyl-CoA-binding protein, PM34: seed maturation protein PM34, GELP: GDSL esterase/lipase. *represents a significant difference in protein expression (fold change ≥ 1.5 and corrected *P* *<* *0.05*)

Seven fatty acid metabolism- and seed development-related proteins (KAS3A, FATA, ALDH2B7, ACBP, PM34, oleosin, and GELP) were selected to validate the results obtained by IBT. The results indicated that while these proteins were detected throughout the different stages of seed development, their levels of abundance varied considerably at different developmental stages compared with the levels present in the 25 DAP samples (Fig. 7). Among the 56 comparisons that were conducted, similar results were evident between the MRM and IBT analyses for 39 (70%) of the proteins. Due to the higher sensitivity of the MRM method in detection and its higher accuracy in quantification^51^, 17 additional comparisons that exhibited a significant fold change were identified. Seven proteins exhibited a bell curve pattern of expression over the course of seed development. KAS3A, FATA, ACBP, and GELP reached a plateau in abundance at 53–67 DAP, ALDH2B7 at 39–53 DAP, and PM34 and oleosin at 67–81 DAP. As expected, the β-galactosidase control was expressed evenly across all eight time points.

#### Correlation analysis of the transcriptome and proteome

**Fig. 8:**
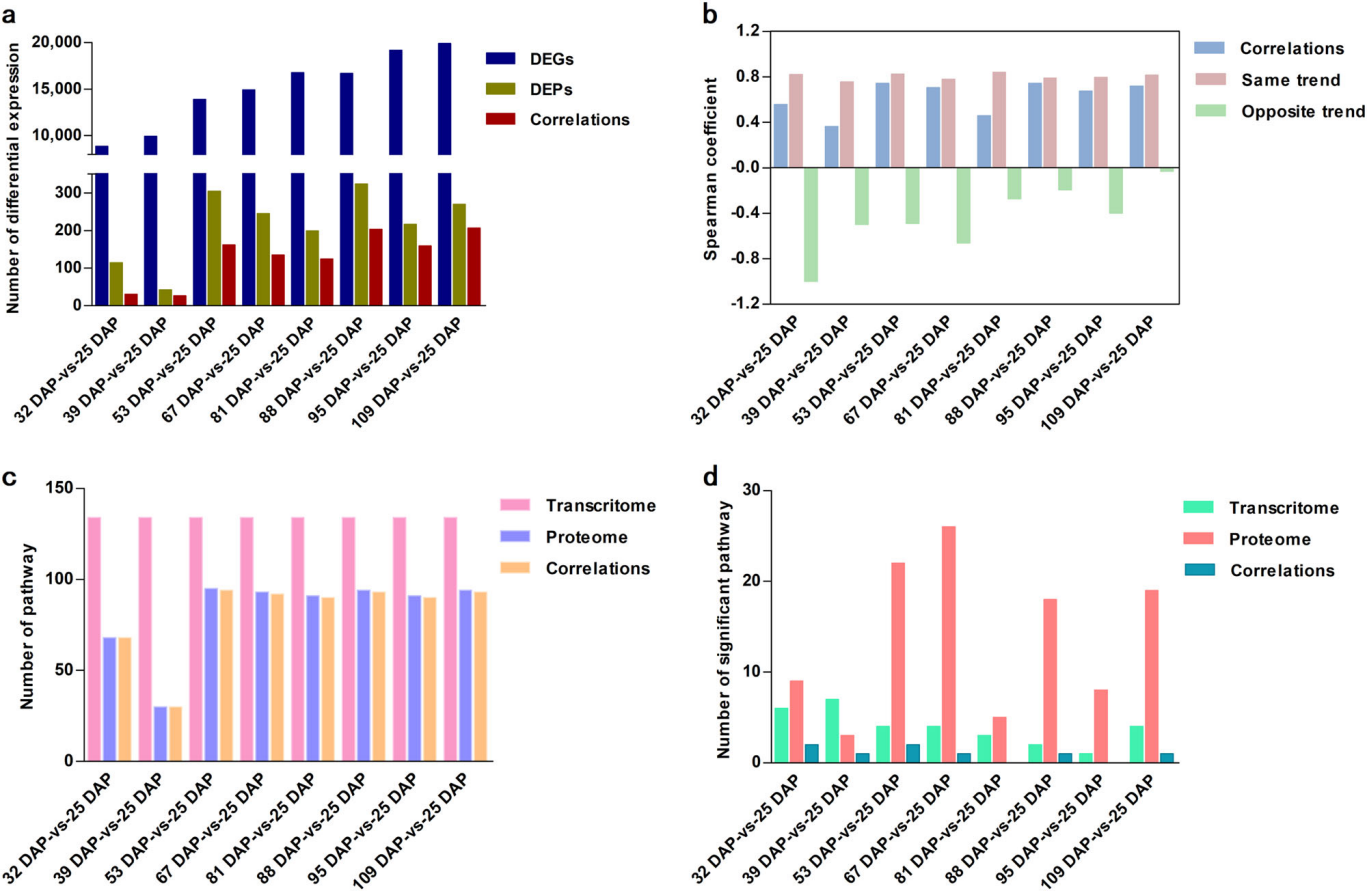
Correlation analysis of the transcriptome and proteome. **a** Correlation analysis of DEG and DEP numbers. **b** Correlation coefficients between the expression levels of correlated DEGs and DEPs. **c** The numbers of all pathways identified in the transcriptome and proteome and their correlations. **d** The numbers of significant pathways identified in the transcriptome and proteome and their correlations

The two different kinds of -omics data were integrated and analyzed to better understand the relationship between the transcriptome and proteome during the development of tree peony seeds. The number of DEGs and DEPs varied greatly, and only a few differentially expressed correlations were observed at different time points (Fig. 8a, Supplementary Table S7). The correlation coefficient between the expression level of correlated DEGs and DEPs ranged from 0.3623 to 0.7439 (in blue). The correlation coefficient of DEGs and DEPs that exhibited a similar pattern of expression was obviously higher than that of DEGs and DEPs that exhibited opposite trends in expression, except at 25 DAP (Fig. 8b), indicating that the expression of correlated DEGs and DEPs exhibits a high level of uniformity at the levels of both transcription and protein abundance. Cluster analysis of the correlated DEGs and DEPs at different developmental time points also revealed consistency in their patterns of expression (Supplementary Fig. S5).

Almost all the KEGG pathways identified in the proteome were correlated with the pathways identified in the transcriptome (Fig. 8c); however, a few correlated pathways were especially enriched (Fig. 8d). These included the flavonoid biosynthesis, ribosome, brassinosteroid biosynthesis, starch and sucrose metabolism, fatty acid biosynthesis, and carbon metabolism pathways (Table 3). The fatty acid biosynthesis pathway was only enriched at 53 DAP according to the transcriptome data, whereas this pathway was enriched at 53, 67, 88 and 109 DAP according to the proteome data. This phenomenon most likely reflects the time delay between transcription and translation.

**Table 3:** Significantly enriched KEGG pathways of correlations in *P. ostii* seeds from different developmental periods

Pathway	Pathway
**32 DAP-vs- 25 DAP**	**81 DAP-vs- 25 DAP**
Flavonoid biosynthesis	NA
Ribosome	
**39 DAP-vs- 25 DAP**	**88 DAP-vs- 25 DAP**
Brassinosteroid biosynthesis	Flavonoid biosynthesis
**53 DAP-vs- 25 DAP**	**95 DAP-vs- 25 DAP**
Starch and sucrose metabolism	NA
Flavonoid biosynthesis	
Fatty acid biosynthesis	**109 DAP-vs- 25 DAP**
**67 DAP-vs- 25 DAP**	Carbon metabolism
Flavonoid biosynthesis	

NA indicates no identified pathway.

## Discussion

Tree peony is an emerging oil crop for the production of ALA. Elucidation of the key stages of seed development and the molecular mechanism of oil synthesis is a critical step that is necessary for the future improvement of tree peony oil in terms of both quantity and quality. The present study provides comprehensive genomic data characterizing gene expression and protein abundance during seed development in the tree peony *P. ostii*, which has a long history of cultivation and exhibits a high seed yield and oil content. In a study conducted by Li et al.^21^ only three time points (30, 60, and 90 DAP) during seed development were investigated in *P. ostii*. In contrast, the present study investigated nine time points (25, 32, 39, 53, 67, 81, 88, 95, 109 DAP) and generated 38,482 unigenes, which greatly increases the available genomic data for tree peony. A total of 26,912 DEGs with a fold change ≥ 2 were identified. The number of DEGs increased as seeds developed and matured compared to the earliest stage (25 DAP) investigated. Our results revealed a greater number of downregulated genes than upregulated genes, which is consistent with the pattern of expression of DEGs reported for soybean seed development^52^. The 211 unigenes putatively associated with fatty acid metabolism, 63 with UFA biosynthesis, and 115 with ALA metabolism should be targeted for future study. A total of 7026 peptides and 2841 proteins were also identified. The current study provides the first large-scale proteomic profile of tree peony seeds, along with a comprehensive analysis of seed development at the level of both the transcriptome and proteome.

*P. ostii* begins to blossom in early April. After pollination, the embryo develops rapidly and reaches maturity in ~100 days. Li et al.^21^ and Ma et al.^23^ classified 25–60 DAP as a rapid growth stage in tree peony seeds based on seed morphology and nutrient content, 60–100 DAP as a seed inclusion enrichment and conversion stage, and 100–115 DAP as a dehydration and maturation stage for both pods and seeds. The nine time points sampled in the current study were chosen based on this classification. The clustering of *P. ostii* DEGs and DEPs generated three similar stages, although the 53 and 67 DAP time points were grouped in the second stage by DEPs and the first stage by DEGs. As previously stated, this difference in the grouped time points most likely reflects the time lapse between transcription and translation. The results of the DEG and DEP analyses conducted in our study corroborated the accumulation of nutrients during *P. ostii* seed development reported by Li et al.^21^ and Ma et al.^23^. For example, at the mature stage (after 100 DAP), when seed changes color from yellow to black (Fig. 1), dehydration occurs; the pods and seeds begin to separate; and the level of fatty acids decreases^23^. The results of our study indicate that this stage exhibited the most uniquely expressed genes (215 vs < 29 at other time points) and the highest number of DEGs. The DEGs and DEPs identified at this stage were enriched for degradation complexes (proteasome, peptidase, and endopeptidase complexes) and carbon metabolism, along with posttranslational modification pathways (N-glycan biosynthesis, glycosylphosphatidylinositol (GPI)-anchor biosynthesis).

**Fig. 9:**
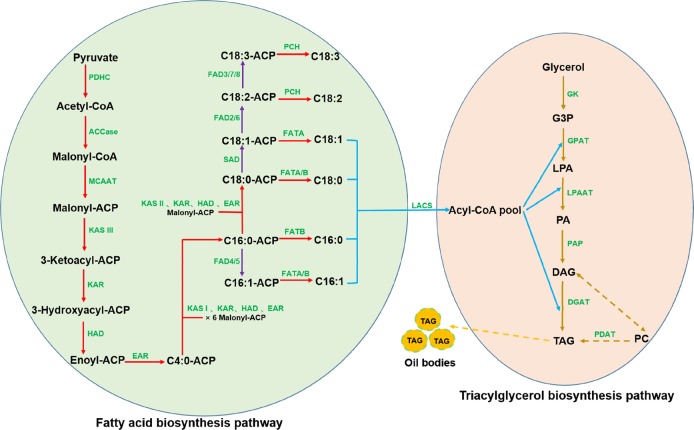
Oil synthesis pathway in *P*. *ostii* seeds. Abbreviations: C16:0, palmitic acid; C16:1, hexadecenoic acid; C18:0, stearic acid; C18:1, oleic acid; C18:2, linoleic acid; C18:3, linolenic acid; ACP, acyl carrier protein; G3P, sn-glycerol-3-phosphate; LPA, lysophosphatidic acid; PA, phosphatidic acid; DAG, sn-1,2-diacylglycerol; TAG, triacylglycerol; PC, phosphatidylcholine; PDHC, pyruvate dehydrogenase; ACCase, acetyl-CoA carboxylase; MCAAT, malonyl-CoA ACP transacylase; KAS I, II, III, ketoacyl-ACP synthase I, II, III; KAR, ketoacyl-ACP reductase; HAD, hydroxyacyl-ACP dehydrase; EAR, enoyl-ACP reductase; SAD, stearoyl-ACP desaturase; FAD2/6, oleate desaturase; FAD3/7/8, linoleate desaturase; FATA/B, acyl-ACP thioesterase A/B; PCH, palmitoyl-CoA hydrolase; LACS, long-chain acyl-CoA synthetase; GK, glycerol kinase; GPAT, glycerol-3-phosphate acyltransferase; LPAAT, 1-acylglycerol-3-phosphate acyltransferase; PAP, phosphatidic acid phosphatase; DGAT, acyl-CoA:diacylglycerol acyltransferase; PDAT, phospholipid:diacylglycerol acyltransferase

Oil synthesis in oil seed plants includes de novo fatty acid biosynthesis, TAG assembly, and oil body formation^27^. Oil synthesis in tree peony seeds appears to follow the same steps based on the results obtained in the present study (Fig. 9) and those reported by Li et al.^23^. First, acetyl-CoA carboxylase (ACCase) catalyzes the formation of the intermediate compound malonyl-CoA from acetyl-CoA. Subsequently, a series of condensation reactions involving malonyl-ACP and a growing acyl-ACP chain are catalyzed by fatty acid synthase subunits, consecutively adding two carbon units per cycle to form 16:0-ACP or 18:0-ACP. After this, free FAs are released from the acyl carrier protein under the control of acyl-ACP thioesterase (FAT) to generate an acyl-CoA pool. TAG is synthesized through sequential acyl-CoA-dependent acylation of a glycerol backbone beginning with sn-glycerol-3-phosphate (G3P). Diacylglycerol acyltransferase (DGAT) then catalyzes a final acylation reaction to form TAG. Alternatively, TAG can be formed via an acyl-CoA-independent pathway, catalyzed by phospholipid:diacylglycerol acyltransferase (PDAT), where phosphatidylcholine (PC) is utilized as an acyl donor in TAG formation. Finally, TAG is combined with oleosin to form an oil body. Transcriptome analysis of seeds from yellow horn (*Xanthoceras sorbifolia* Bunge)^27^, another woody oil crop, identified an oil biosynthesis pathway almost identical to that of tree peony. The same mechanisms regulating oil biosynthesis have been reported in transcriptome and proteome studies of oilseed rape, an herbaceous oil crop^26^. The collective data from these studies and the current study indicate that the process of oil synthesis among oilseed plants is very similar.

KEGG pathway enrichment analysis of DEGs indicated that genes encoding components of fatty acid biosynthesis pathways were enriched at 53 DAP. DEPs associated with fatty acid biosynthesis were enriched at 53~67 DAP, 88 DAP, and 109 DAP. In particular, two isoforms encoding *PDAT*, a key mediator of the synthesis of plant TAG, reached their highest expression level at 67 DAP, as determined by both RNA-seq and RT-qPCR analyses (Fig. 5). TAG, as a storage form of fat energy, consists of three fatty acids and serves as an energy reserve for germination and postgermination growth. Fan et al.^53^ reported that overexpression of *PDAT* stimulates fatty acid and TAG synthesis, and coexpression of *PDAT* with oleosin increases the dry weight content of triacylglycerol by up to 6.4%. In the current study, MRM analysis of seven proteins that play an important role in fatty acid metabolism and seed development revealed high abundance during the period of time in which *P. ostii* seeds are undergoing rapid development. KAS3A initiates elongation in type II fatty acid synthase systems using acetyl-CoA as the primer and malonyl-ACP as the acceptor. Acyl-ACP thioesterases play an essential role in chain termination during fatty acid synthesis and exhibit two distinct classes (FATA and FATB). FATA presents higher specificity for 18:1-ACP, while FATB prefers acyl-ACPs with saturated acyl groups^54^. The ALDH superfamily in plants is a group of NAD(P)^+^-dependent enzymes that catalyze the oxidation of aromatic and aliphatic aldehydes into their corresponding carboxylic acids, including fatty acids, and reduce lipid peroxidation. Shin et al.^55^ identified an ALDH in rice that is necessary for seed maturation and seed viability. ALDH2 proteins produce acetate from acetaldehyde, which is used for acetyl-CoA biosynthesis^56^. ACBP is a highly conserved 10 kDa protein that exclusively binds to long-chain fatty acyl-CoAs. Ablation of ACBP can induce preimplantation embryonic lethality in mice^57^. Chen et al.^58^ reported that ACBP1 and ACBP2 in *Arabidopsis* are highly expressed during seed development and that the *acbp1acbp2* double mutant is embryonic lethal. ACBP1 is also reported to negatively modulate sterol synthesis during embryogenesis.

The seed maturation protein PM34 specifically accumulates in the seeds of several species, including *Medicago truncatula*, alfalfa, soybean, and rice. While the exact function of PM34 in seeds has not been demonstrated, Sano et al.^59^ suggested that this protein plays a role in rice seed quality. Satour et al.^60^ found that a PM34 in *M. truncatula* exhibits cellulase activity and high sensitivity to carbonylation. GELPs are a very large and important subfamily of lipolytic enzymes that exhibit biochemical activity involved in the regulation of development, synthesis of secondary metabolites, and defense responses. There are 108, 96, 130, 126, 105, 121, and 114 *GELP* genes in *Arabidopsis thaliana*, *Vitis vinifera*, *Sorghum bicolour*, *Populus trichocarpa, Brassica napa*, and rice, respectively^61–64^. Ectopic overexpression of a cotton *GELP* gene in *Arabidopsis* plants increased seed length and fresh weight as well as the level of soluble sugars and storage proteins, suggesting its involvement in ovule and fiber development^65^. Among the 108 *GELP* genes found in *Arabidopsis*, 45 exhibit expression in seeds and other tissue types, and two are exclusively expressed in seeds^64^.

Oleosins are small proteins that cover the surface of subcellular lipid storage bodies (oil bodies) in seeds. Fan et al.^53^ reported that overexpression of oleosin promotes the clustering of small oil droplets in *Arabidopsis* seeds. The results of our current study indicate that 53–88 DAP is a period of rapid fatty acid biosynthesis in *P. ostii* seeds, which is consistent with the findings of Xiu et al.^66^.

Fatty acid desaturases (FADs) convert saturated fatty acids into UFAs by removing two hydrogen atoms from a fatty acid and creating a carbon/carbon double bond (Fig. 9). FAD are classified into delta (at the carboxyl end of a fatty acid) or omega (at the methyl end of a fatty acid) desaturases depending on where the carbon/carbon double bond occurs. These enzymes are highly substrate specific. For instance, FAB2, also known as stearoyl-ACP desaturase (SAD), converts stearic acid (18:0) to oleic acid (18:1) in a acyl-carrier protein (ACP)-bound form. FAD2 and FAD6 are ω-6 desaturases that synthesize the dienoic fatty acid linoleic acid (18:2) from oleic acid (18:1). FAD3, FAD7, and FAD8 are ω-3 desaturases that insert a double bond in linoleic acid (18:2) to synthesize linolenic acid (18:3). FAD4 and FAD5 act specifically on palmitic acid (16:0) to produce palmitoleic acid (16:1)^38^. *FAD* genes have been identified in several oilseed crops, including soybean^67^, cacao^68^ and olive^69^. *FAD3* in olive is primarily responsible for the 18:3 contents of seeds, while two *FAD7* genes contribute primarily to the 18:3 contents of the mesocarp of fruits^69^. In the current study, all 16 of the annotated *FADs* exhibited differential expression during seed development, suggesting that they play a significant role in converting saturated fatty acids into UFAs in tree peony seeds. More than 90% of the total fatty acids in tree peony seeds are unsaturated, and ALA accounts for approximately 45% of the total content of unsaturated fatty acids^7^.

ALA (18:3) can be biosynthesized from saturated fatty acids (18:0), monounsaturated fatty acids (18:1), and diunsaturated fatty acids (18:2) via three desaturation reactions catalyzed by SAD, FAD2, and FAD3, respectively. The three genes encoding these proteins were reported to exhibit their highest expression in tree peony (*P. suffruticosa*) at 56 or 70 DAP depending on the cultivar, with *PsFAD3* exhibiting a much higher expression level than the other two genes^39^. Transgenic *Arabidopsis* seeds expressing *PsFAD3* contain more ALA than wild-type seeds, while linoleic acid (18:2) is converted into ALA by PsFAD3 in transgenic yeast^39^. Zhang et al.^70^ also reported that *PrFAD2* and *PrFAD3* increased linoleic and ALA contents, respectively, in *P. rockii*. In our study, eight *FAD* unigenes (CL4788. Contig1_All, CL952. Contig1_All, CL952. Contig2_All, Unigene22956_All, CL952. Contig3_All, CL4788. Contig4_All, CL12028. Contig3_All and Unigene23379_All) exhibited their highest expression at 53 DAP, which is similar to the findings reported by Yin et al.^39^. In particular, the unigene CL12028. Contig3_All exhibited an expression level that was at least 3.5 times higher than the others, suggesting that it plays an important role in ALA accumulation in *P. ostii*. These eight genes may play a similar role in the biosynthesis of ALA since they exhibit the same expression pattern. Interestingly, fatty acid metabolism and degradation pathways were enriched at the same four time points at which DEP fatty acid metabolism was enriched. This may suggest that as fatty acids are being synthesized, they are also utilized to generate derived products such as triacylglycerols and UFAs.

According to the central dogma of molecular biology, protein-coding genes are transcribed into messenger RNAs, which are then translated into proteins. Proteins may be further processed by posttranslational modifications. While direct correspondence is generally assumed to exist between mRNA transcripts and protein abundance, recent studies have reported that the correlation between these two parameters is actually low due to a variety of factors, including variable half-lives and posttranscriptional regulation of mRNAs^24^. Our proteomic data largely validated the transcriptomic results, as demonstrated by the commonly enriched KEGG pathways and the similar expression patterns of DEGs and DEPs in the correlation analysis. The changes in transcript levels did not, however, always correspond to similar changes in protein levels. For example, the KAS3A, FATA, and ACBP proteins exhibited high levels of abundance at 53 and 67 DAP; however, their corresponding transcripts were not differentially expressed (Fig. 7). Fatty acid biosynthesis-associated DEGs were only enriched at 53 DAP, while fatty acid biosynthesis DEPs were enriched at 53~67 DAP, 88 DAP, and 109 DAP. These findings suggest that seed development and oil synthesis are regulated at multiple levels. Therefore, a combined transcriptome and proteome analysis can provide unique insights.

In summary, a total of 38,482 unigenes and 2,841 proteins were identified from nine developmental time points in tree peony seeds (*P. ostii*). The samples from all of the time points together clustered in two groups based on their correlation coefficients and sample distances. The number of condition-specific genes was greatest at seed maturation (109 DAP). The development of tree peony seeds was grouped into three developmental stages based on the cluster analysis of 26,912 DEGs and 592 DEPs. The three stages were rapid growth, seed inclusion enrichment and conversion, and late dehydration and maturation. Fifteen lipid metabolism pathways were also identified in both the transcriptome and proteome data, and a period of rapid fatty acid biosynthesis was identified at 53–88 DAP. In total, 211 genes and 35 proteins associated with fatty acid metabolism were identified, along with 63 genes and 11 proteins associated with UFA biosynthesis, and 115 genes and 24 proteins associated with ALA metabolism. Notably, phylogenetic analysis revealed 16 putative FAD-encoding genes that clustered in four *FAD* groups. Eight of the *FAD* genes exhibited their highest expression at 53 DAP, suggesting that they play an important role in ALA accumulation. Overall, the RT-qPCR analysis of the temporal expression pattern of oil biosynthesis genes was largely in agreement with the RNA-seq results. The patterns of the abundance of fatty acid metabolism- and seed development-related proteins determined by MRM were highly consistent with the results obtained in the proteomic analysis. The integrated analysis of the two kinds of -omics data (transcriptomic and proteomic) revealed that there were significant differences between the number and abundance of DEGs vs. DEPs, but a high level of similarity in expression patterns and enrichment of specific metabolic pathways were also observed. The present study provides a wealth of new transcriptomic and proteomic data on seed development in tree peony that can be used for developing strategies to improve the yield and quality of tree peony seed oil.

## Supplementary information

Supplementary Table and Supplementary Figure.
