# Supplementary material for: Integrated analysis of transcriptomic and proteomic data from tree peony (P. ostii) seeds reveals key developmental stages and candidate genes related to oil biosynthesis and fatty acid metabolism
Source: Hortic Res. 2019 Oct 1;6:111. doi: 10.1038/s41438-019-0194-7 (PMC6804530; doi:10.1038/s41438-019-0194-7)

Integrated analysis of transcriptomic and proteomic data from tree peony (*P. ostii*) seeds reveals key developmental stages and candidate genes related to oil biosynthesis and fatty acid metabolism

Xiaojing Wang [1], Haiying Liang [2], Dalong Guo[3], Lili Guo [1], Xiangguang Duan [1], Qishi Jia [1], Xiaogai Hou [1*]

Supporting information

Table S1. Summary of sequencing data of *P. ostii* seeds and alignment information of clean reads.

Sample	Raw Data Size (bp)	Raw Data Number	Clean Data Number	Clean Data Rate (%)	Total Mapped Reads (%)	Unique Match (%)	Multi-position Match (%)
25 DAP_1	1206863350	24137264	24114971	99.90	59.22	42.11	17.11
25 DAP_2	1206863350	24136880	24116796	99.91	59.47	42.29	17.18
32 DAP_1	1206863350	24137240	24040658	99.59	63.43	45.39	18.03
32 DAP_2	1206863350	24137468	24019317	99.51	63.01	45.14	17.87
39 DAP_1	1206863350	24137118	24078445	99.75	56.24	39.62	16.63
39 DAP_2	1206863350	24137409	24055346	99.66	57.53	40.50	17.02
53 DAP_1	1206863350	24137222	24092972	99.81	51.10	39.55	11.56
53 DAP_2	1206863350	24137299	24112567	99.89	51.03	39.50	11.53
67 DAP_1	1206863350	24137353	24113946	99.90	53.85	42.57	11.28
67 DAP_2	1206863350	24137486	24110018	99.88	53.78	42.50	11.29
81 DAP_1	1206863350	24137221	24113728	99.90	49.13	38.39	10.74
81 DAP_2	1206863350	24137260	24115643	99.10	49.48	38.79	10.69
88 DAP_1	1206846350	24136927	24115340	99.91	47.33	37.97	9.36
88 DAP_2	1206863350	24137267	24115968	99.91	46.46	37.45	9.01
95 DAP_1	1206863350	24137370	24113203	99.89	53.49	42.84	10.64
95 DAP_2	1206863350	24137099	24115207	99.90	53.57	42.93	10.63
109 DAP_1	1206863350	24137291	24045153	99.61	49.03	37.90	11.14
109 DAP_2	1206863350	24137012	24090253	99.80	49.36	37.79	11.57

Table S2. The common unique genes between *P. ostii* seeds from the two peony trees.

Table S2.xlsx

Table S3. Significantly enriched GO biological processes in *P. ostii* seeds of different development period (*P < 0.05*).

**GO biological processes**	**DEGs with GO annotation**	**All genes with GO annotation**	**Corrected P-value**
**32 DAP-vs- 25 DAP**			
Nucleosome organization	49	82	8.89e-12
Protein-DNA complex subunit organization	82	82	8.89e-12
Chromatin organization	54	112	1.11e-07
Macromolecular complex subunit organization	67	185	0.00049
**53 DAP-vs- 25 DAP**			
Phenylpropanoid biosynthetic process	14	16	0.01912
Secondary metabolite biosynthetic process	14	16	0.01912
Nucleosome organization	47	82	0.03219
Protein-DNA complex subunit organization	47	82	0.03219
**81 DAP-vs- 25 DAP**			
Nucleosome organization	23	43	0.0014
Protein-DNA complex subunit organization	49	119	0.0014
**88 DAP-vs- 25 DAP**			
Nucleosome organization	60	82	0.0016
Protein-DNA complex subunit organization	60	82	0.0016
**95 DAP-vs- 25 DAP**			
Metabolic process	3308	6200	0.01279

Significantly enriched GO cellular components in *P. ostii* seeds of different development period (*P < 0.05*).

**GO cellular components**	**DEGs with GO annotation**	**All genes with GO annotation**	**Corrected P-value**
**32 DAP-vs- 25 DAP**			
Chromatin	43	73	8.79e-10
Chromosomal part	49	108	5.25e-06
Chromosome	51	115	6.99e-06
Thylakoid	47	114	0.00030
Plastid	136	444	0.00073
Intracellular membrane-bounded organelle	414	1653	0.02279
Non-membrane-bounded organelle	66	202	0.02904
Intracellular non-membrane-bounded organelle	66	202	0.02904

**39 DAP-vs- 25 DAP**			
Intrinsic component of membrane	288	1051	0.00118
Membrane part	322	1215	0.00688
Membrane	420	1650	0.02134
Endoplasmic reticulum	15	28	0.03381
**53 DAP-vs- 25 DAP**			
Thylakoid	62	114	0.00105
**67 DAP-vs- 25 DAP**			
Thylakoid	63	114	0.03368
**81 DAP-vs- 25 DAP**			
Chromatin	51	73	0.00116
Mitochondrial part	45	63	0.00161
Mitochondrion	46	66	0.00363
Mitochondrial envelope	39	55	0.00832
**88 DAP-vs- 25 DAP**			
Intracellular ribonucleoprotein complex	281	470	2.89e-09
Ribonucleoprotein complex	281	470	2.89e-09
Macromolecular complex	557	1087	0.00057
Chromatin	52	73	0.00083
**95 DAP-vs- 25 DAP**			
Intracellular ribonucleoprotein complex	325	470	1.37e-12
Ribonucleoprotein complex	325	470	1.37e-12
Macromolecular complex	639	1087	0.00020
**109 DAP-vs- 25 DAP**			
Proteasome complex	35	44	0.02893
Peptidase complex	35	44	0.02893
Endopeptidase complex	35	44	0.02893

Significantly enriched GO molecular functions in *P. ostii* seeds of different development period ($P < 0.05$).

GO molecular functions	DEGs with GO annotation	All genes with GO annotation	Corrected P-value
**39 DAP-vs- 25 DAP**			
Hydrolase activity, acting on glycosyl bonds	119	329	8.82e-06
Carboxypeptidase activity	20	32	0.00060
Hydrolase activity, hydrolyzing O-glycosyl compounds	81	224	0.00145
Iron ion binding	91	259	0.00153
Oxidoreductase activity	339	1205	0.00183
Beta-glucosidase activity	15	25	0.02384
Glucosidase activity	30	68	0.02828
**67 DAP-vs- 25 DAP**			
Hydrolase activity, acting on glycosyl bonds	160	329	0.04694

**88 DAP-vs- 25 DAP**			
Structural molecule activity	241	399	7.64e-09
**95 DAP-vs- 25 DAP**			
Structural molecule activity	281	399	1.42e-13
**109 DAP-vs- 25 DAP**			
Catalytic activity	3881	7135	1.30e-05
Oxidoreductase activity, acting on CH-OH group of donors	129	194	0.02245

Table S4. All genes and proteins associated with lipid metabolism identified in the transcriptome and proteome.

**Lipid metabolism pathway**	**All genes with pathway annotation (28112)**	**All proteins with pathway annotation (2475)**	**Pathway ID**
Fatty acid metabolism	211 (0.75%)	35 (1.41%)	ko01212
Fatty acid biosynthesis	93 (0.33%)	26 (1.05%)	ko00061
Fatty acid degradation	98 (0.35%)	31 (1.25%)	ko00071
Fatty acid elongation	131 (0.47%)	7 (0.28%)	ko00062
UFA biosynthesis	63 (0.22%)	11 (0.44%)	ko01040
Linoleic acid metabolism	46 (0.16%)	11 (0.44%)	ko00591
ALA metabolism	115 (0.41%)	24 (0.97%)	ko00592
Steroid biosynthesis	80 (0.28%)	8 (0.32%)	ko00100
Sphingolipid metabolism	141 (0.5%)	19 (0.77%)	ko00600
Glycerolipid metabolism	306 (1.09%)	24 (0.97%)	ko00561
Ether lipid metabolism	122 (0.43%)	10 (0.4%)	ko00565
Arachidonic acid metabolism	69 (0.25%)	17 (0.69%)	ko00590
Glycerophospholipid metabolism	306 (1.09%)	27 (1.09%)	ko00564
Cutin, suberine and wax biosynthesis	95 (0.34%)	9 (0.36%)	ko00073
Synthesis and degradation of ketone bodies	18 (0.06%)	1 (0.04%)	ko00072

Table S5. All proteins identified in developing *P. ostii* seeds.

Table S5.xlsx

Table S6. Pathway-based categories of proteins in *P. ostii* seeds.

	**Pathway**	**Count (2475)**	**Pathway ID**
1	Metabolic pathways	821	ko01100
2	Biosynthesis of secondary metabolites	483	ko01110
3	Carbon metabolism	174	ko01200
4	Biosynthesis of amino acids	148	ko01230
5	Protein processing in endoplasmic reticulum	134	ko04141
6	Ribosome	115	ko03010
7	Glycolysis / Gluconeogenesis	93	ko00010
8	Amino sugar and nucleotide sugar metabolism	77	ko00520
9	Endocytosis	76	ko04144
10	Starch and sucrose metabolism	75	ko00500
11	RNA transport	75	ko03013
12	Pyruvate metabolism	64	ko00620
13	Oxidative phosphorylation	55	ko00190
14	Purine metabolism	54	ko00230
15	Spliceosome	54	ko03040
16	Peroxisome	52	ko04146
17	Carbon fixation in photosynthetic organisms	49	ko00710
18	Glutathione metabolism	48	ko00480
19	Citrate cycle (TCA cycle)	47	ko00020
20	Cysteine and methionine metabolism	46	ko00270
21	2-Oxocarboxylic acid metabolism	44	ko01210
22	Phenylpropanoid biosynthesis	43	ko00940
23	Pyrimidine metabolism	42	ko00240
24	Ubiquitin mediated proteolysis	42	ko04120
25	Proteasome	41	ko03050
26	RNA degradation	41	ko03018
27	Glyoxylate and dicarboxylate metabolism	40	ko00630
28	Plant-pathogen interaction	38	ko04626
29	Pentose phosphate pathway	37	ko00030
30	Phagosome	36	ko04145
31	Galactose metabolism	35	ko000052
32	Fatty acid metabolism	35	ko01212
33	Fructose and mannose metabolism	34	ko00051
34	mRNA surveillance pathway	33	ko03015
35	Tyrosine metabolism	33	ko00350
36	Aminoacyl-tRNA biosynthesis	32	ko00970
37	Fatty acid degradation	31	ko00071
38	Ascorbate and aldarate metabolism	31	ko00053

39	MAPK signaling pathway - plant	30	ko04016
40	Glycine, serine and threonine metabolism	30	ko00260
41	Alanine, aspartate and glutamate metabolism	29	ko00250
42	Glycerophospholipid metabolism	27	ko00564
43	Pentose and glucuronate interconversions	27	ko00040
44	Arginine and proline metabolism	27	ko00330
45	Fatty acid biosynthesis	26	ko00061
46	Inositol phosphate metabolism	25	ko00562
47	Terpenoid backbone biosynthesis	25	ko00900
48	Phenylalanine, tyrosine and tryptophan biosynthesis	25	ko00400
49	Valine, leucine and isoleucine degradation	24	ko00280
50	ALA metabolism	24	ko00592
51	Glycerolipid metabolism	24	ko00561
52	Pantothenate and CoA biosynthesis	23	ko00770
53	Phenylalanine metabolism	23	ko00360
54	Flavonoid biosynthesis	22	ko00941
55	Propanoate metabolism	22	ko00640
56	Other glycan degradation	22	ko00511
57	Cyanoamino acid metabolism	22	ko00460
58	Protein export	21	ko03060
59	Ubiquinone and other terpenoid-quinone biosynthesis	21	ko00130
60	N-Glycan biosynthesis	20	ko00510
61	Arginine biosynthesis	20	ko00220
62	Tryptophan metabolism	20	ko00380
63	Phosphatidylinositol signaling system	20	ko04070
64	Sphingolipid metabolism	19	ko00600
65	Ribosome biogenesis in eukaryotes	18	ko03008
66	beta-Alanine metabolism	17	ko00410
67	Plant hormone signal transduction	17	ko04075
68	Arachidonic acid metabolism	17	ko00590
69	Valine, leucine and isoleucine biosynthesis	15	ko00290
70	Porphyrin and chlorophyll metabolism	14	ko00860
71	Nucleotide excision repair	14	ko03420
72	One carbon pool by folate	14	ko00670
73	Vitamin B6 metabolism	14	ko00750
74	Tropane, piperidine and pyridine alkaloid biosynthesis	13	ko00960
75	Glycosphingolipid biosynthesis - ganglio series	12	ko00604
76	Selenocompound metabolism	12	ko00450
77	Glycosaminoglycan degradation	12	ko00531
78	Butanoate metabolism	12	ko00650

79	SNARE interactions in vesicular transport	12	ko04130
80	Linoleic acid metabolism	11	ko00591
81	UFA biosynthesis	11	ko01040
82	Isoquinoline alkaloid biosynthesis	11	ko00950
83	Sulfur metabolism	11	ko00920
84	Nitrogen metabolism	11	ko00910
85	DNA replication	11	ko03030
86	ABC transporters	10	ko02010
87	Lysine degradation	10	ko00310
88	Ether lipid metabolism	10	ko00565
89	Thiamine metabolism	10	ko00730
90	Isoflavonoid biosynthesis	9	ko00943
91	Cutin, suberine and wax biosynthesis	9	ko00073
92	Homologous recombination	9	ko03440
93	Mismatch repair	9	ko03430
94	Folate biosynthesis	9	ko00790
95	Nicotinate and nicotinamide metabolism	9	ko00760
96	Biotin metabolism	9	ko00780
97	Histidine metabolism	8	ko00340
98	Steroid biosynthesis	8	ko00100
99	Glycosphingolipid biosynthesis - globo and isoglobo series	8	ko00603
100	RNA polymerase	8	ko03020
101	Stilbenoid, diarylheptanoid and gingerol biosynthesis	7	ko00945
102	Lysine biosynthesis	7	ko00300
103	Riboflavin metabolism	7	ko00740
104	Fatty acid elongation	7	ko00062
105	Other types of O-glycan biosynthesis	7	ko00514
106	Taurine and hypotaurine metabolism	6	ko00430
107	C5-Branched dibasic acid metabolism	6	ko00660
108	Limonene and pinene degradation	5	ko00903
109	Base excision repair	5	ko03410
110	Circadian rhythm - plant	5	ko04712
111	Photosynthesis	5	ko00195
112	Anthocyanin biosynthesis	5	ko00942
113	Carotenoid biosynthesis	5	ko00906
114	Indole alkaloid biosynthesis	5	ko00901
115	Basal transcription factors	3	ko03022
116	Monobactam biosynthesis	3	ko00261
117	Brassinosteroid biosynthesis	3	ko00905
118	Glycosylphosphatidylinositol (GPI)-anchor biosynthesis	3	ko00563

119	Flavone and flavonol biosynthesis	3	ko00944
120	Sulfur relay system	2	ko04122
121	Sesquiterpenoid and triterpenoid biosynthesis	2	ko00909
122	Monoterpenoid biosynthesis	2	ko00902
123	Benzoxazinoid biosynthesis	2	ko00402
124	Zeatin biosynthesis	1	ko00908
125	Photosynthesis - antenna proteins	1	ko00196
126	AGE-RAGE signaling pathway in diabetic complications	1	ko04933
127	Caffeine metabolism	1	ko00232
128	Phosphonate and phosphinate metabolism	1	ko00440
129	Glucosinolate biosynthesis	1	ko00966
130	Non-homologous end-joining	1	ko03450
131	Synthesis and degradation of ketone bodies	1	ko00072
132	Vancomycin resistance	1	ko01502

Table S7. Differential expressed correlations at different time points

Table S7.xlsx

Figure S1. Number of identified unigenes in each *P. ostii* seeds sample.

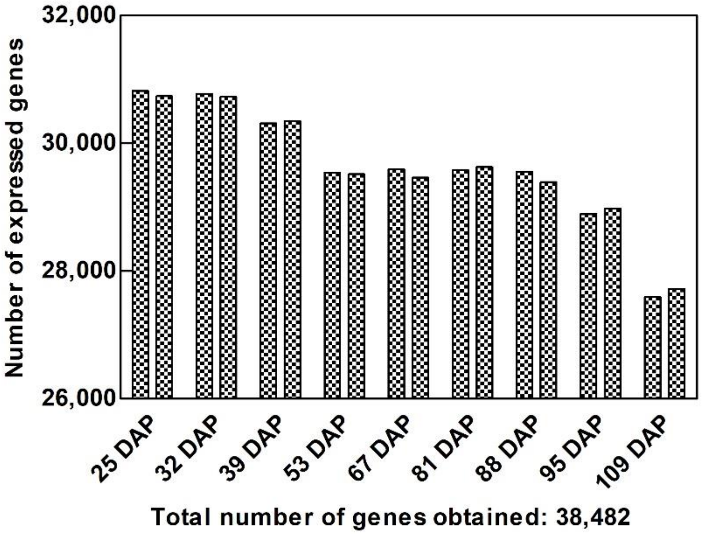

Figure S2. GO functional classification of *P. ostii* DEGs for the eight comparisons.

32 DAP-vs- 25 DAP

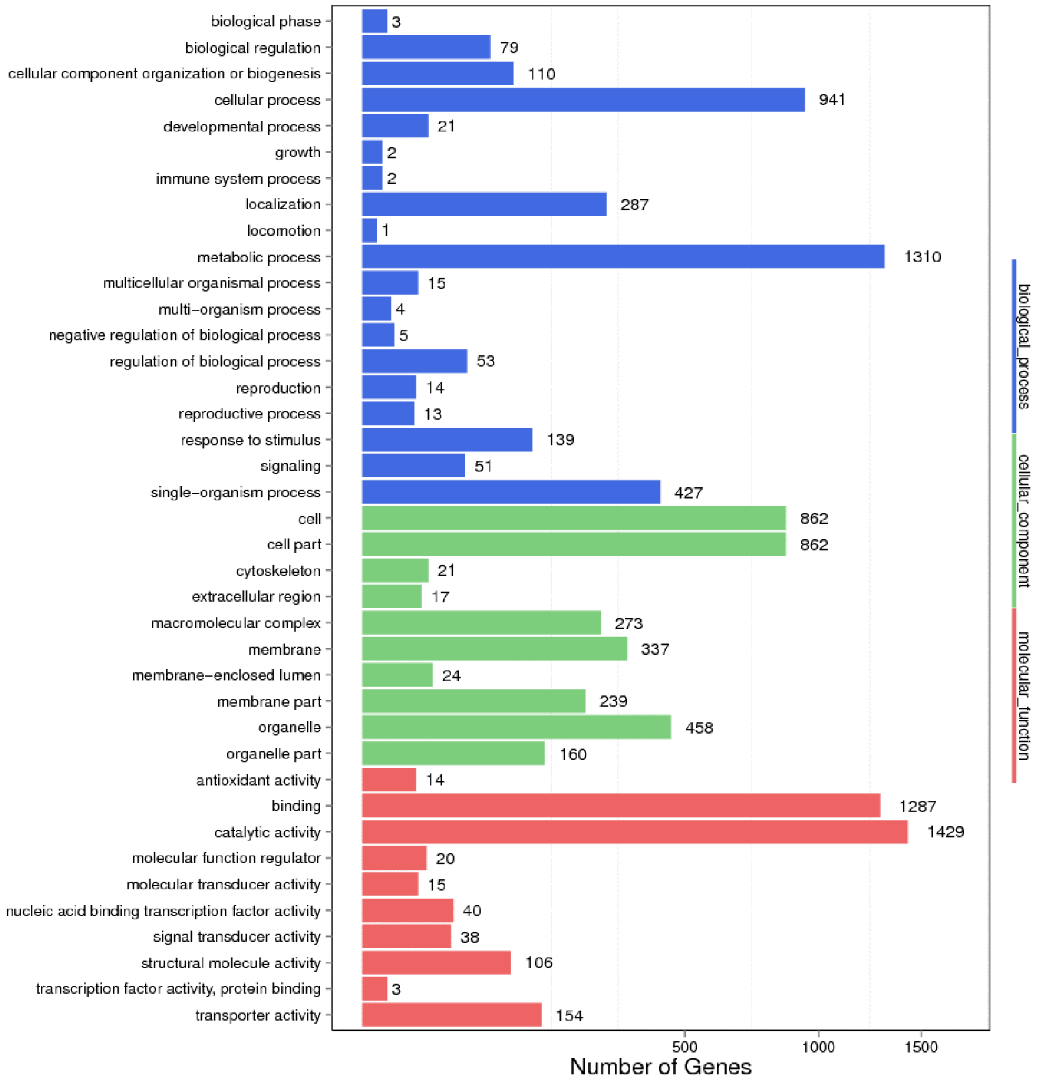

39 DAP-vs- 25 DAP

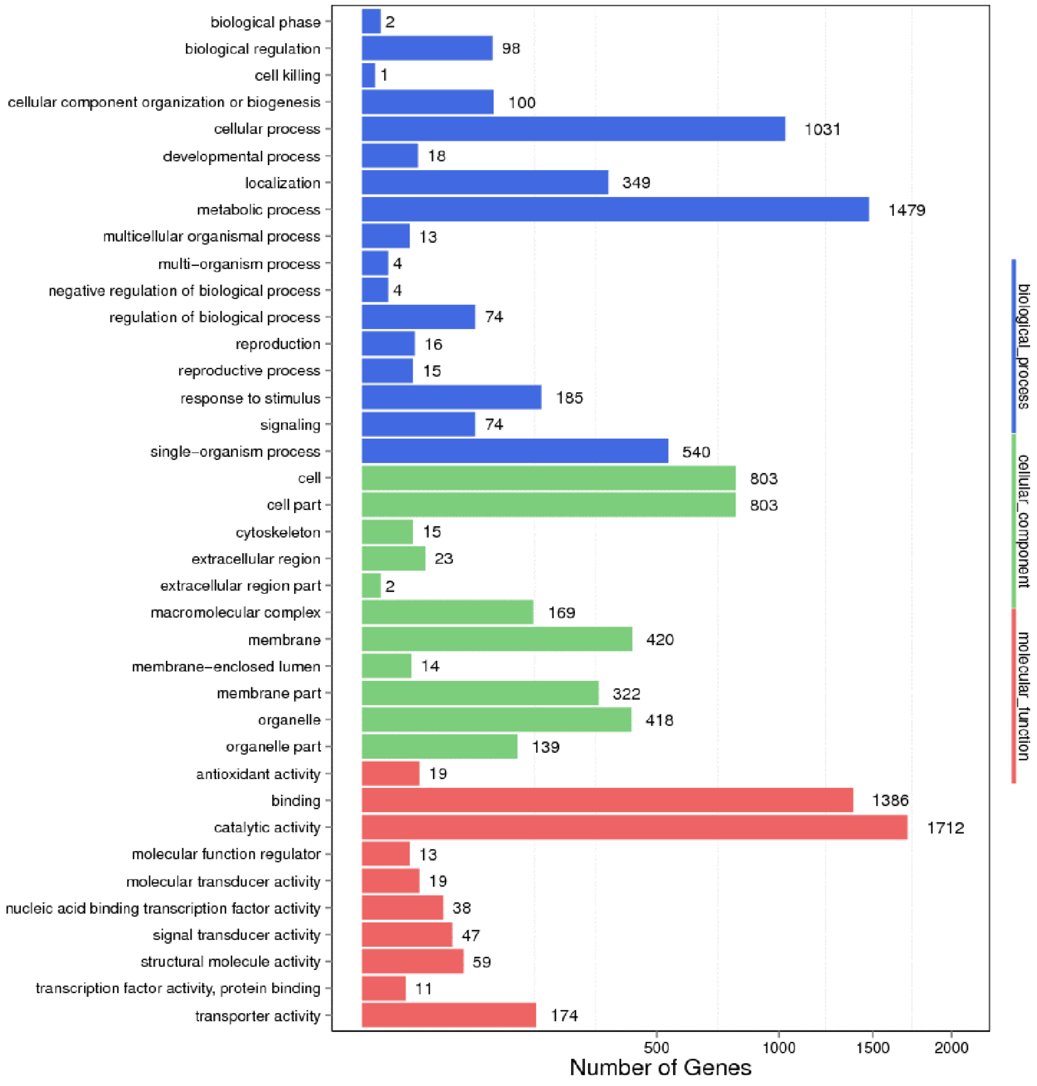

53 DAP-vs- 25 DAP

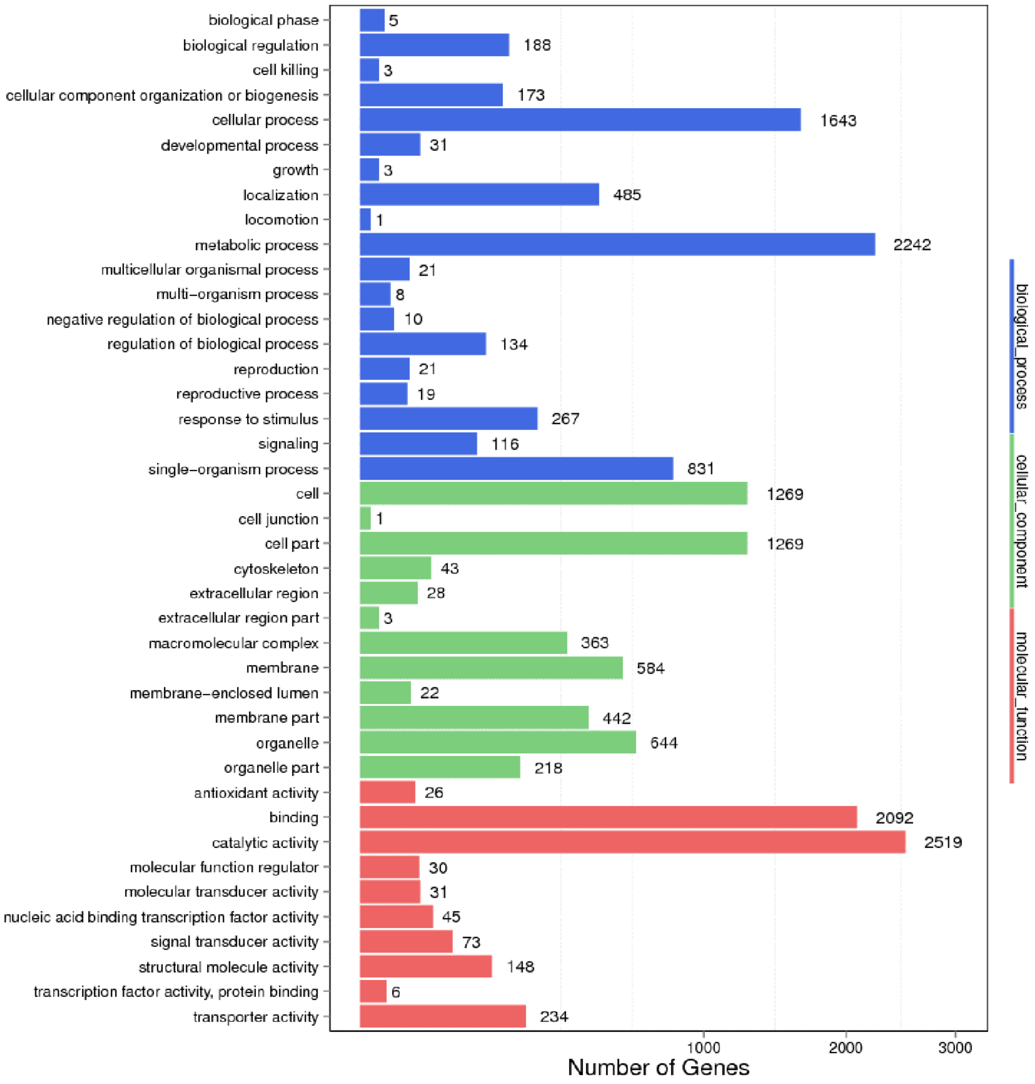

67 DAP-vs- 25 DAP

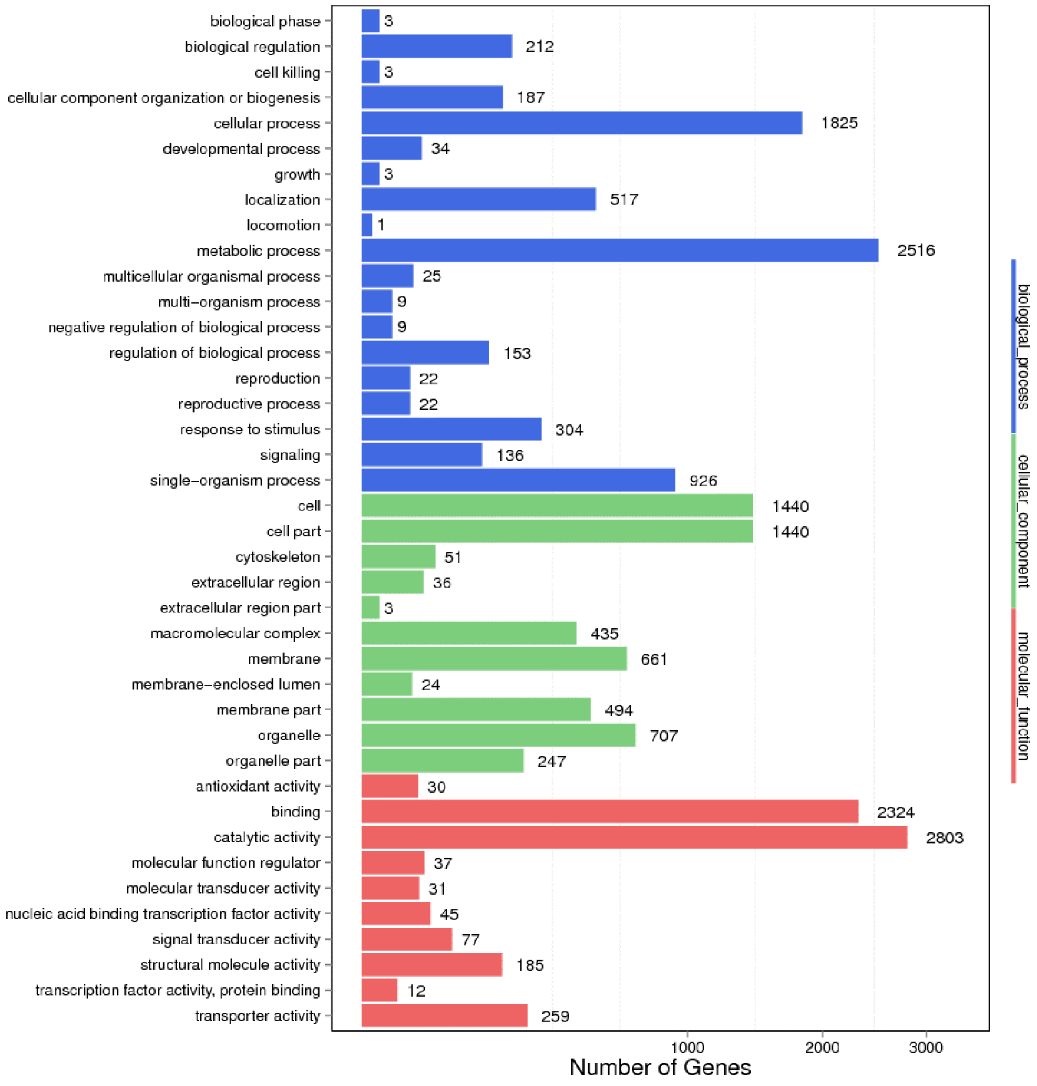

81 DAP-vs- 25 DAP

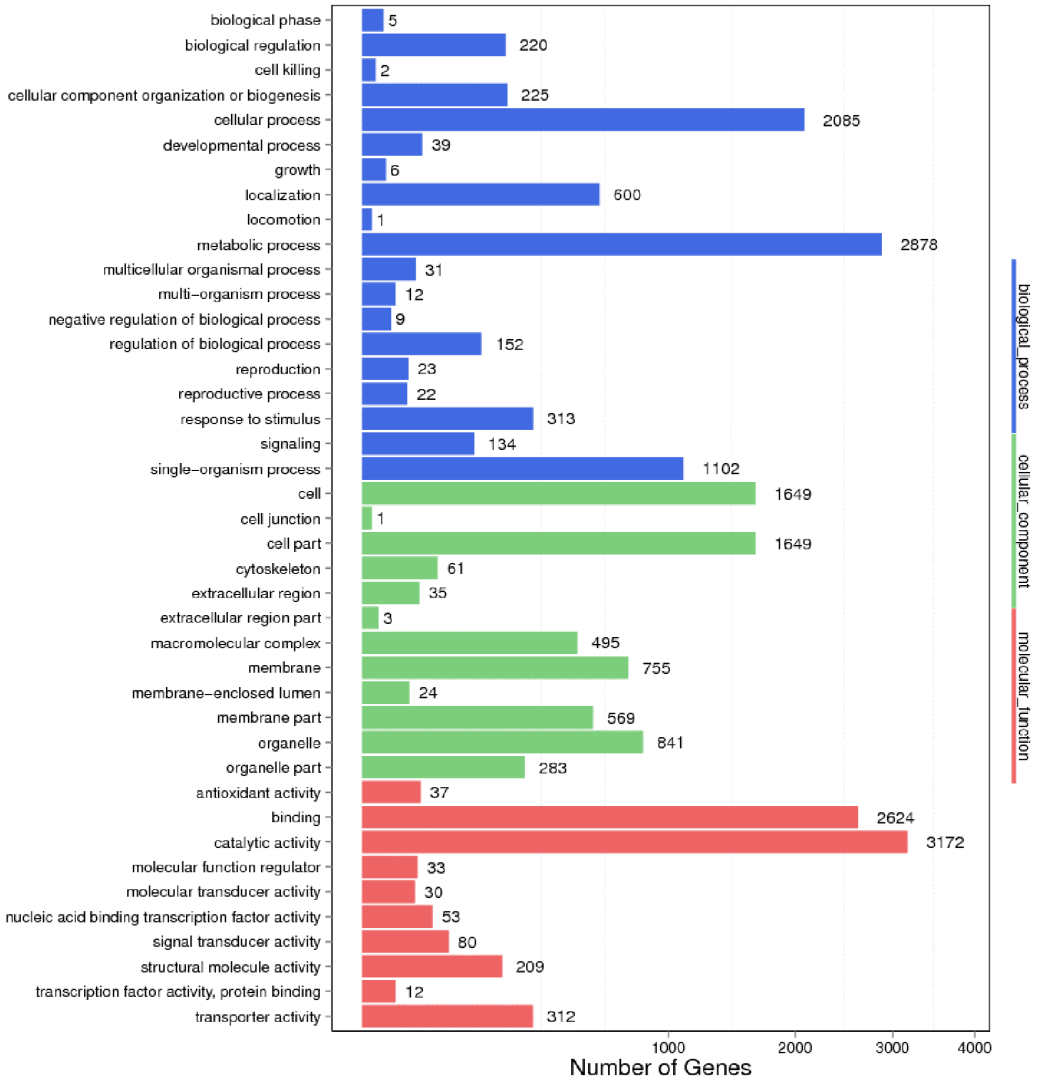

88 DAP-vs- 25 DAP

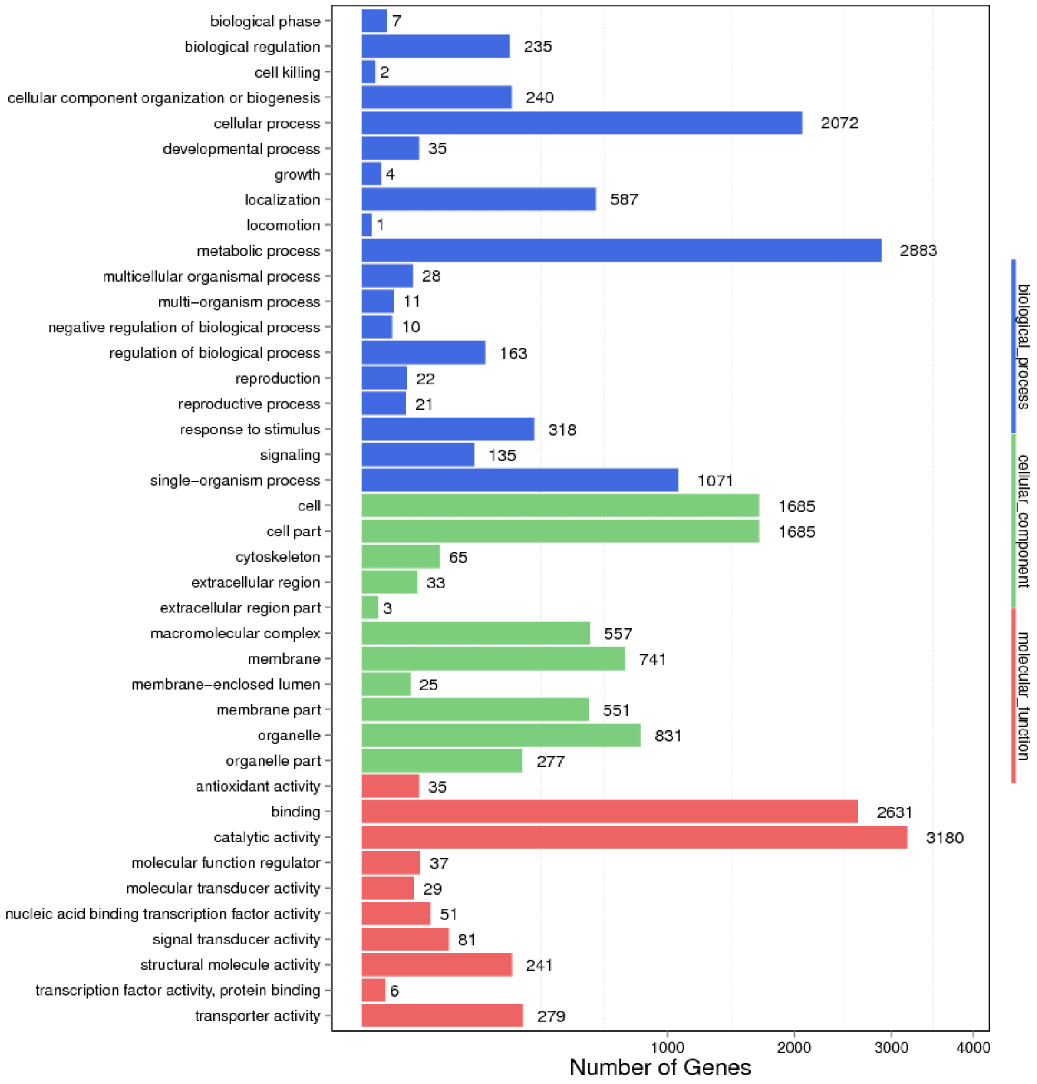

95 DAP-vs- 25 DAP

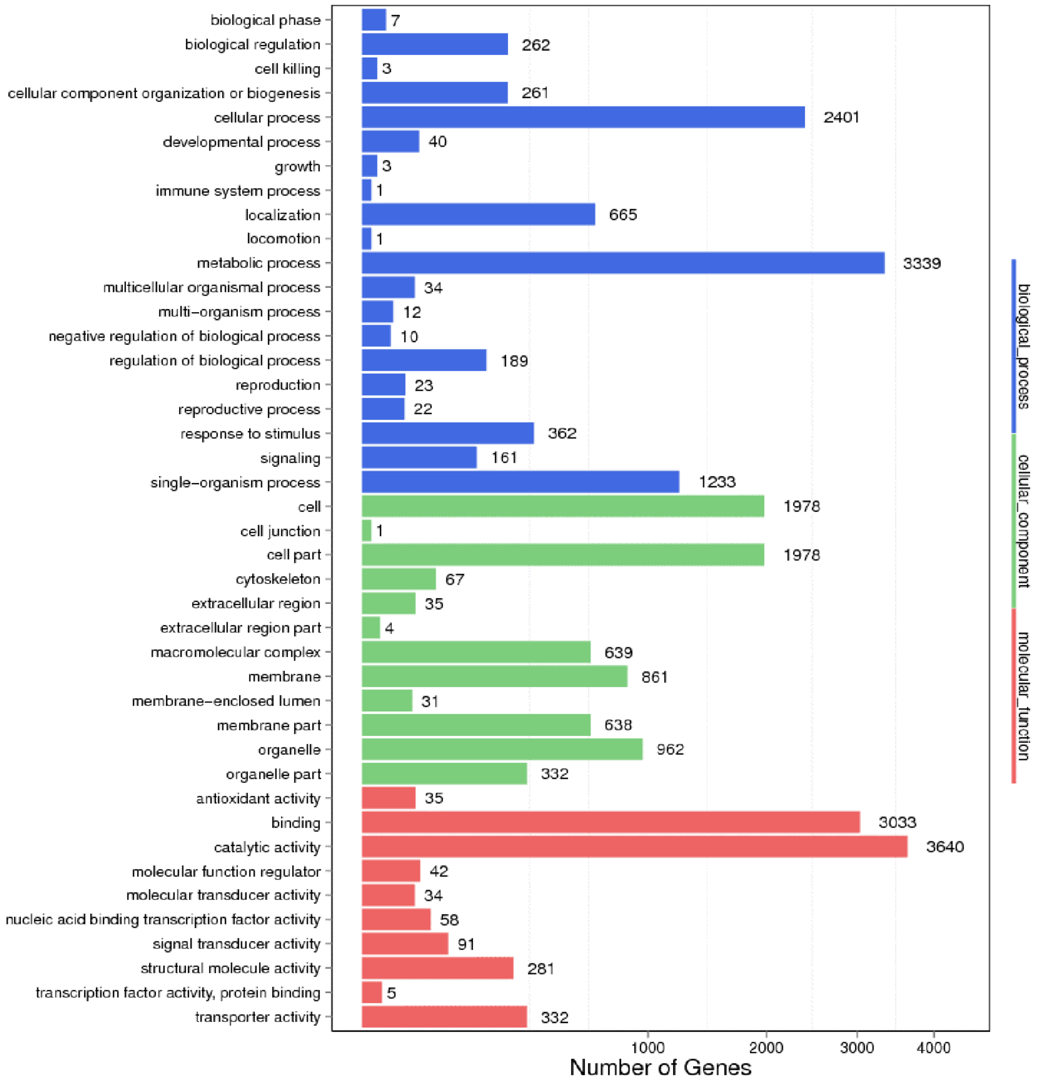

109 DAP-vs- 25 DAP

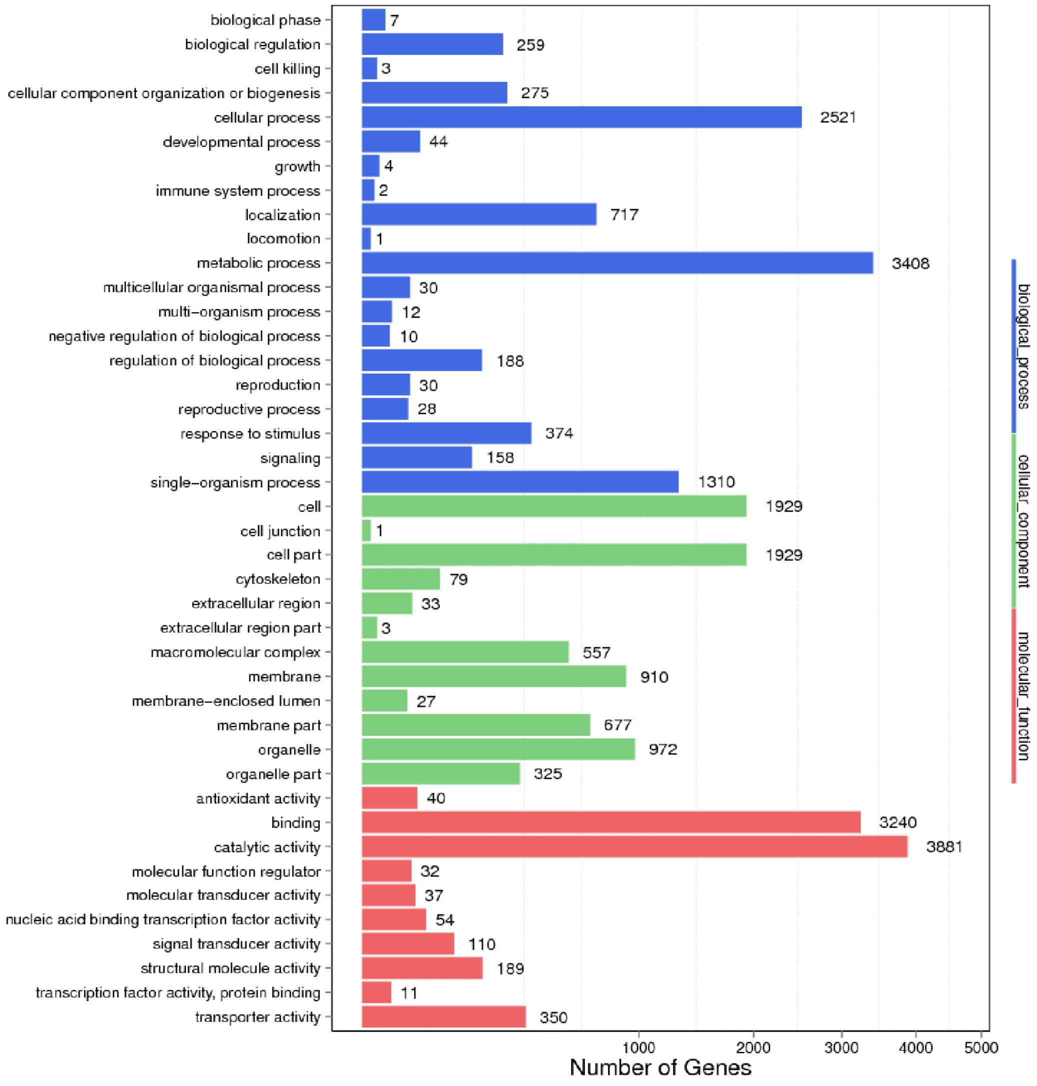

Figure S3. KEGG function classification of DEGs for each comparison.

32 DAP-vs- 25 DAP

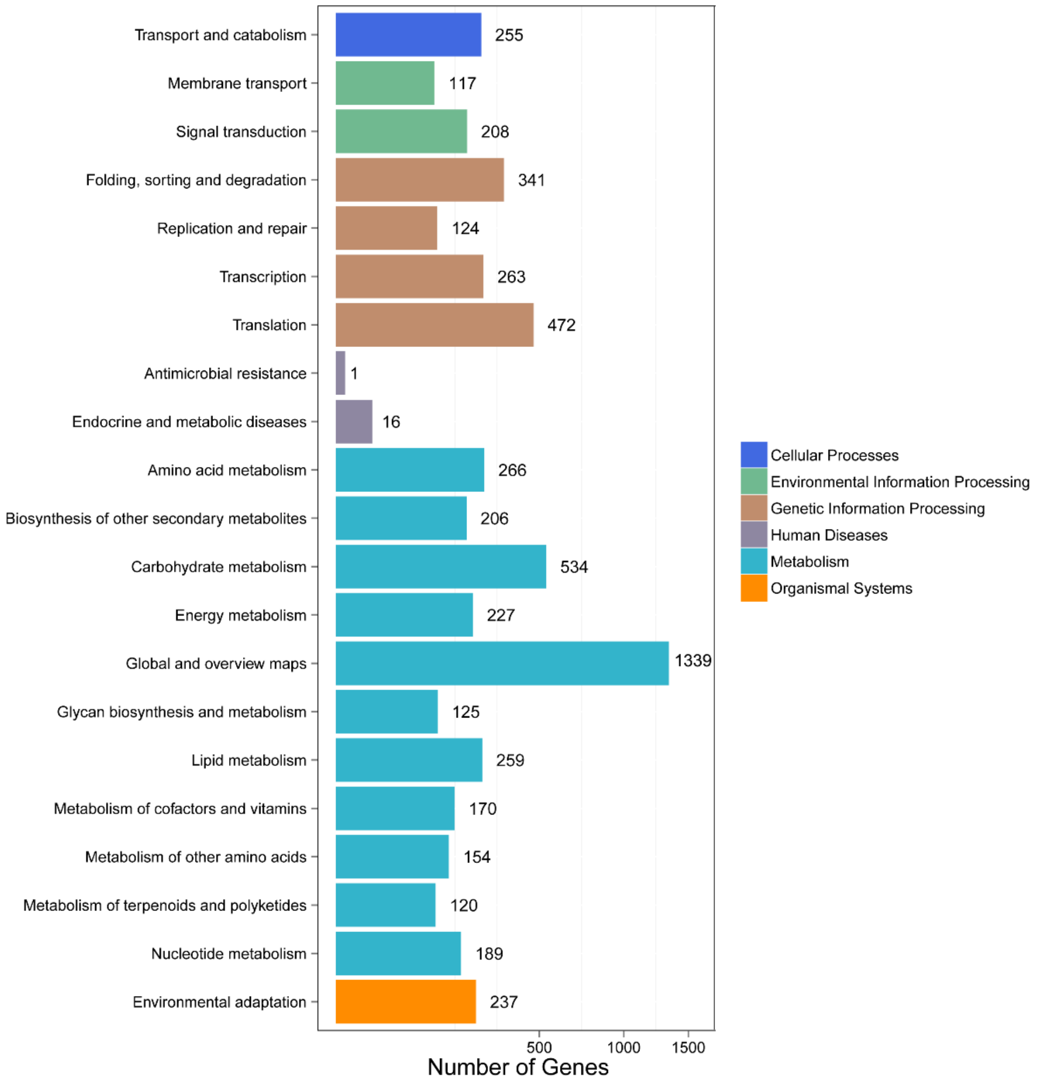

39 DAP-vs- 25 DAP

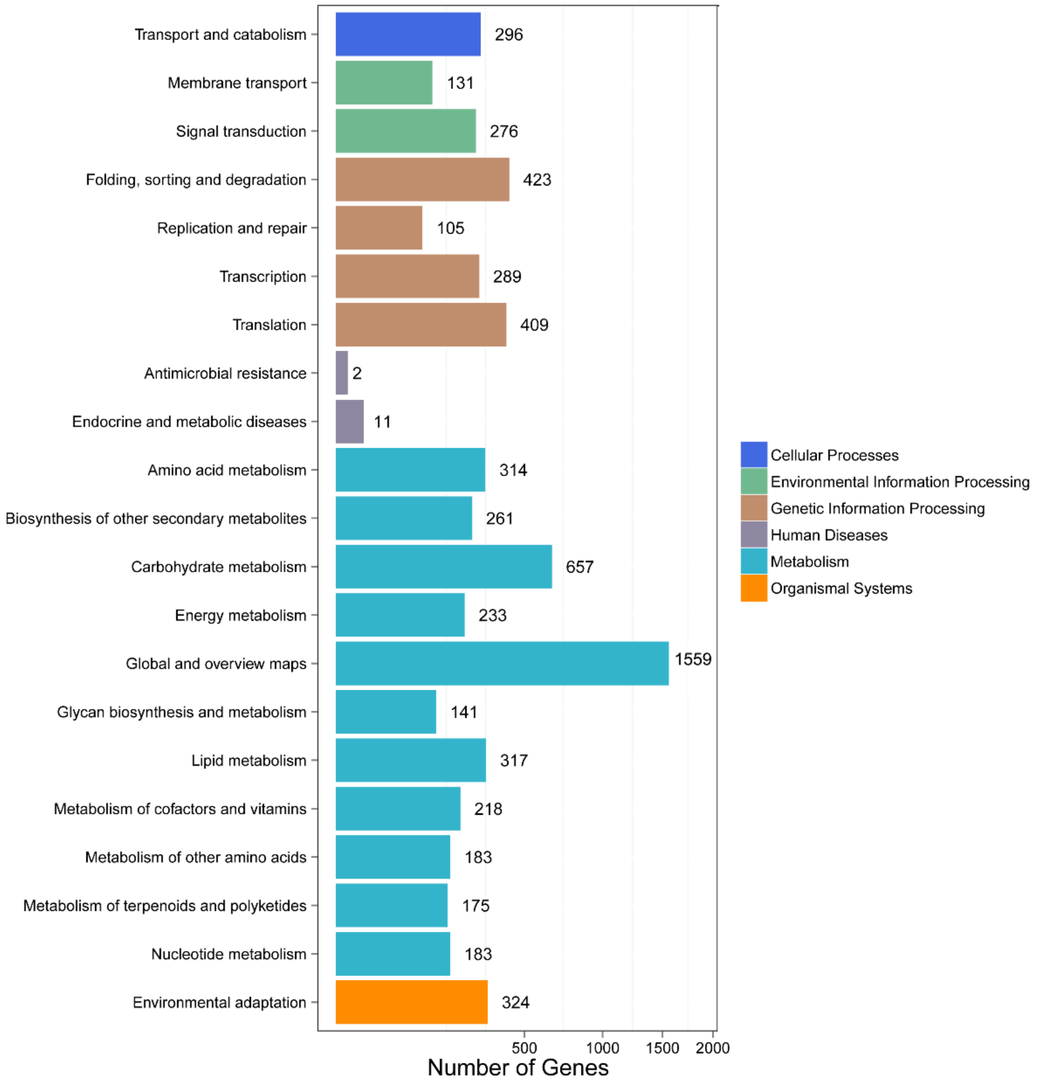

53 DAP-vs- 25 DAP

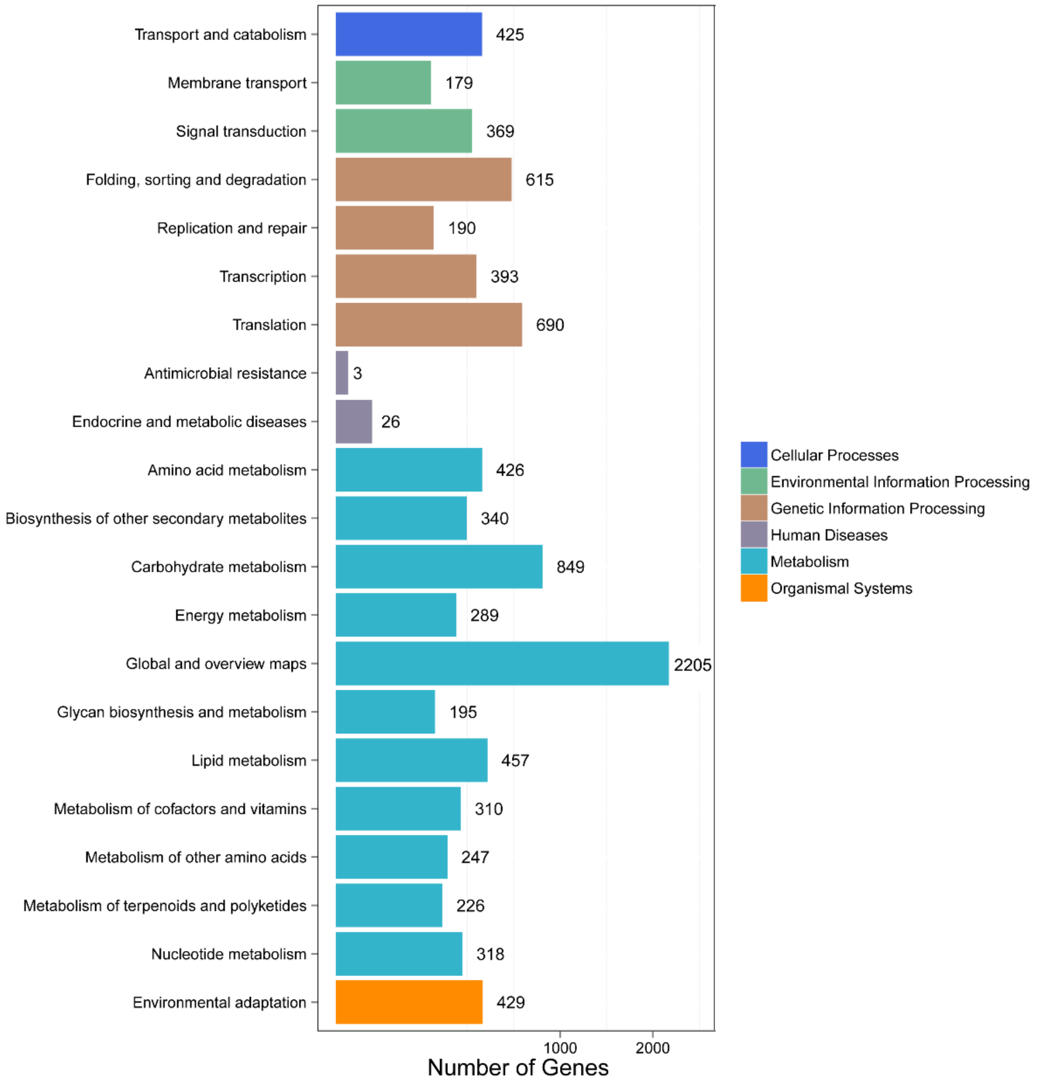

67 DAP-vs- 25 DAP

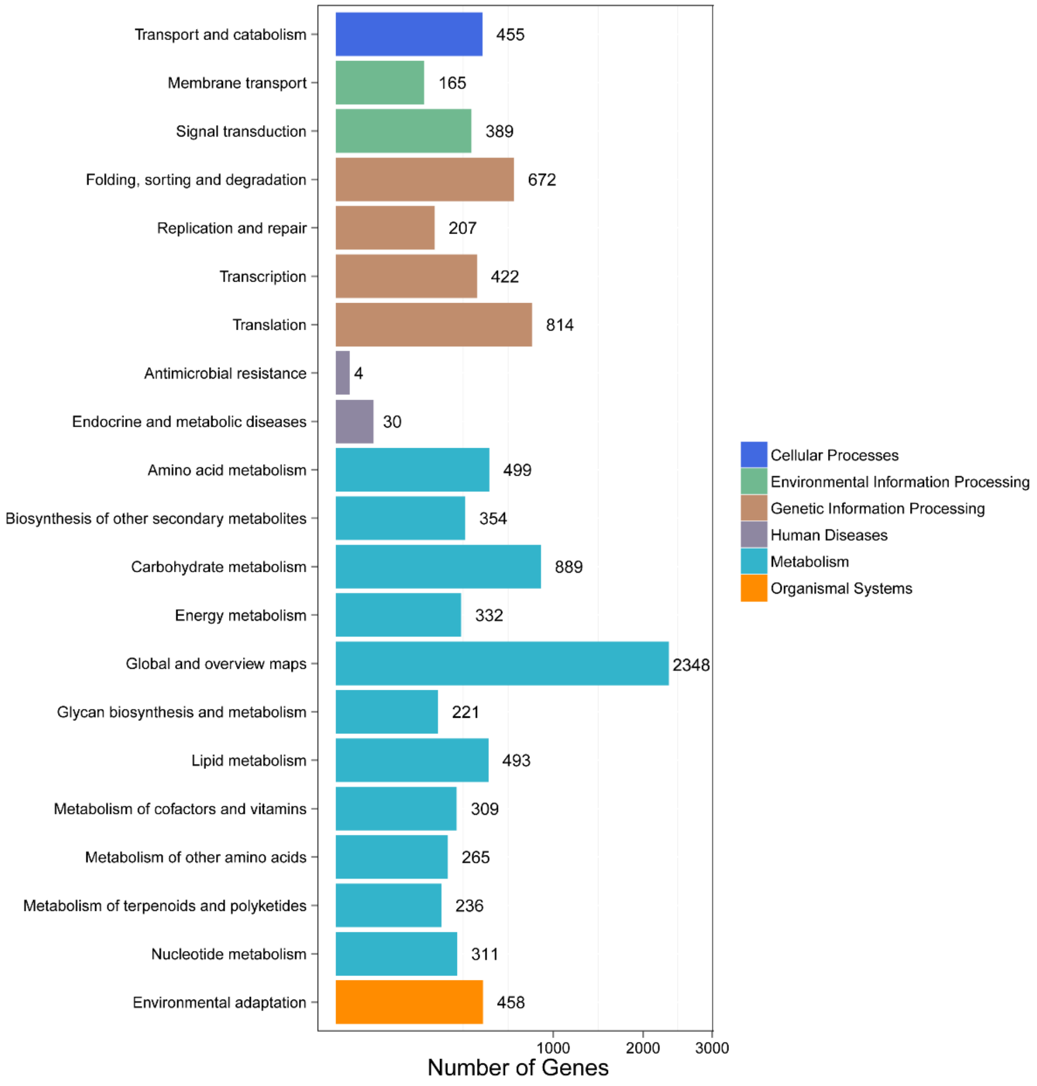

81 DAP-vs- 25 DAP

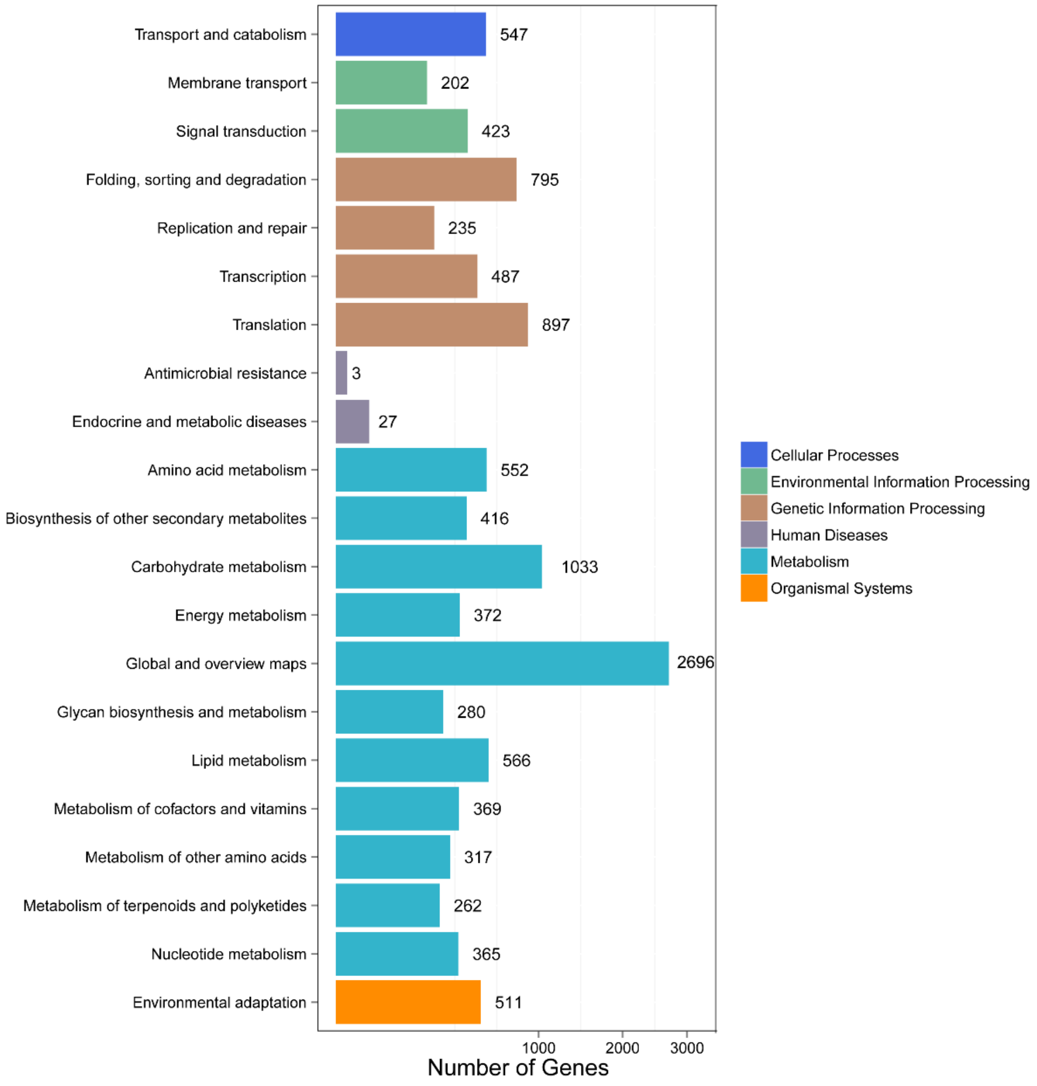

88 DAP-vs- 25 DAP

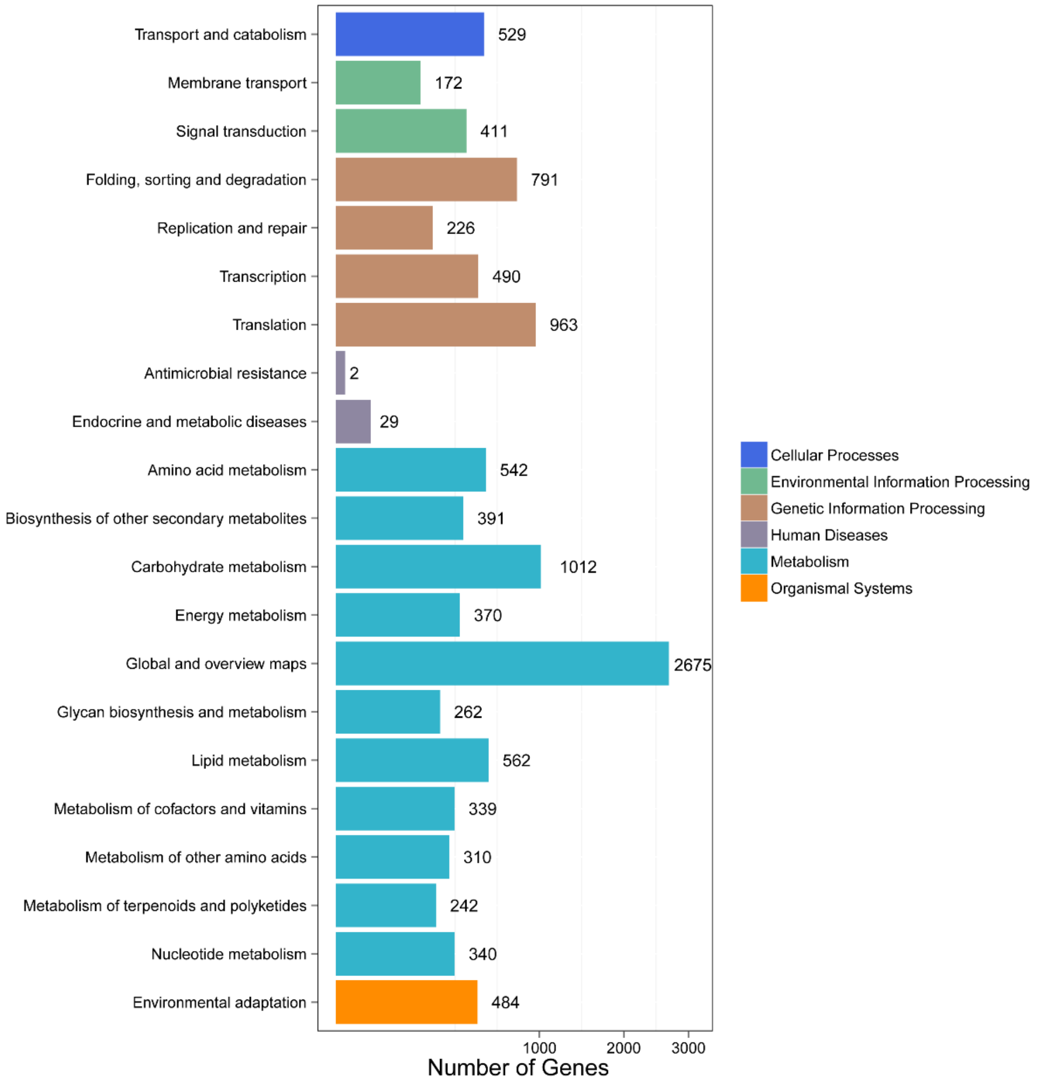

95 DAP-vs- 25 DAP

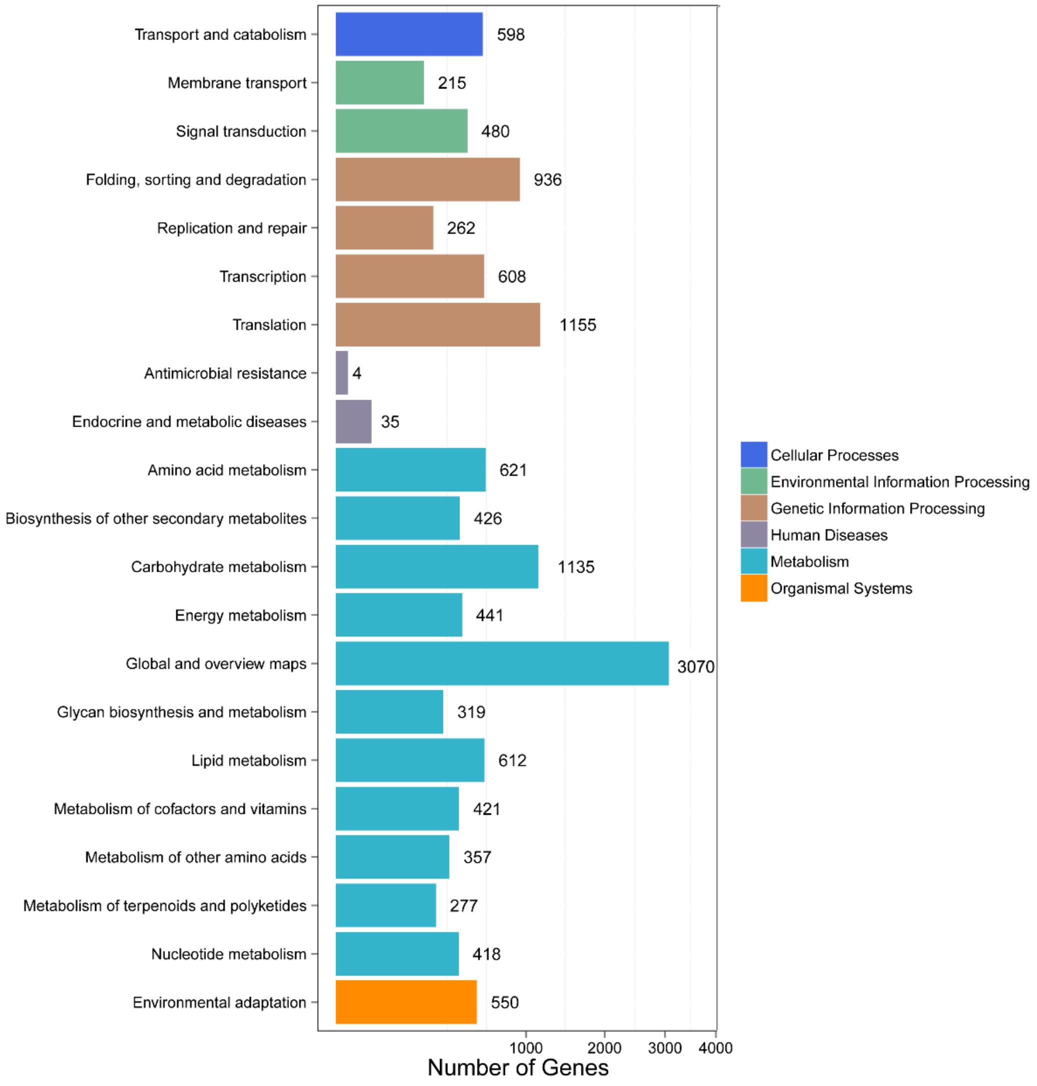

109 DAP-vs- 25 DAP

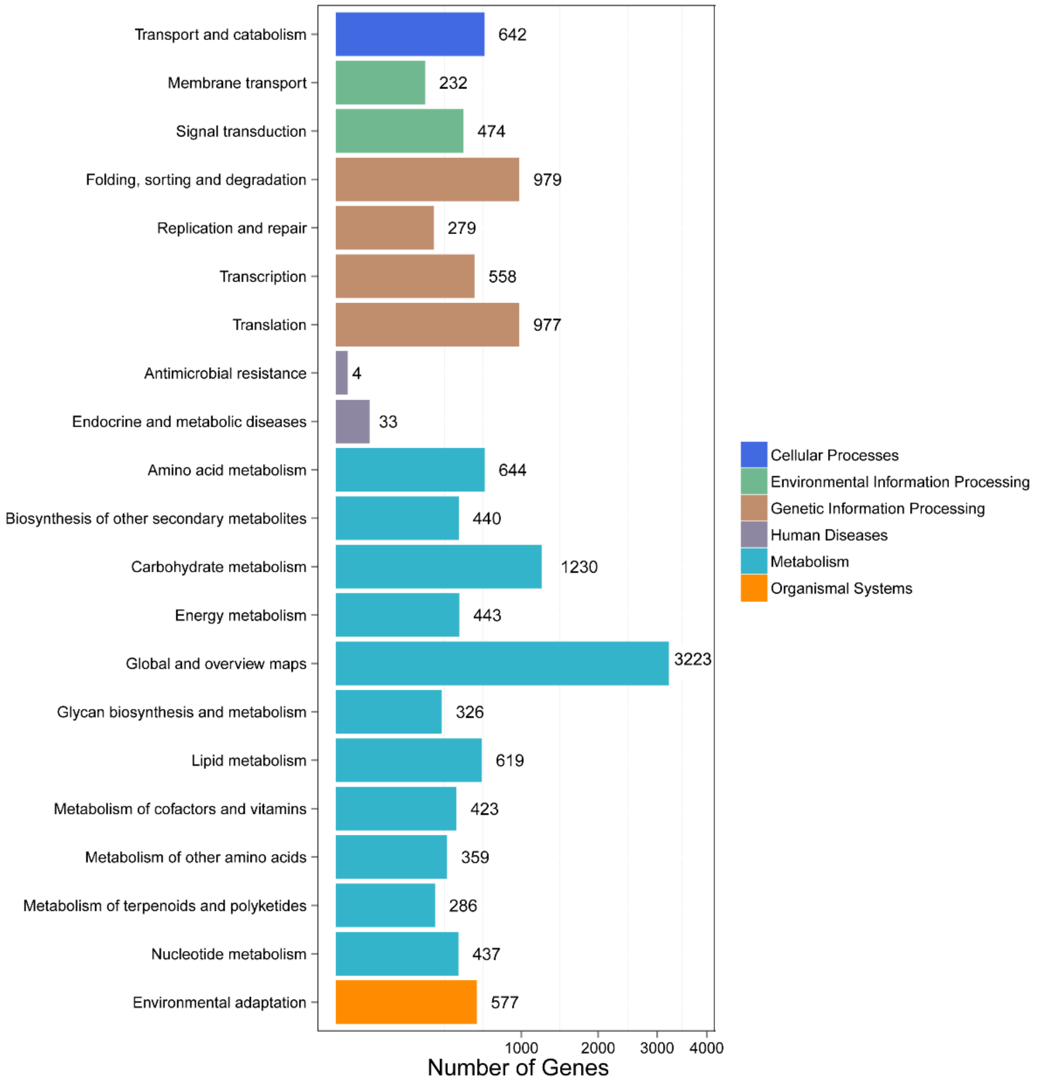

Figure S4. Functional classification of GO annotated proteins in *P. ostii* seeds.

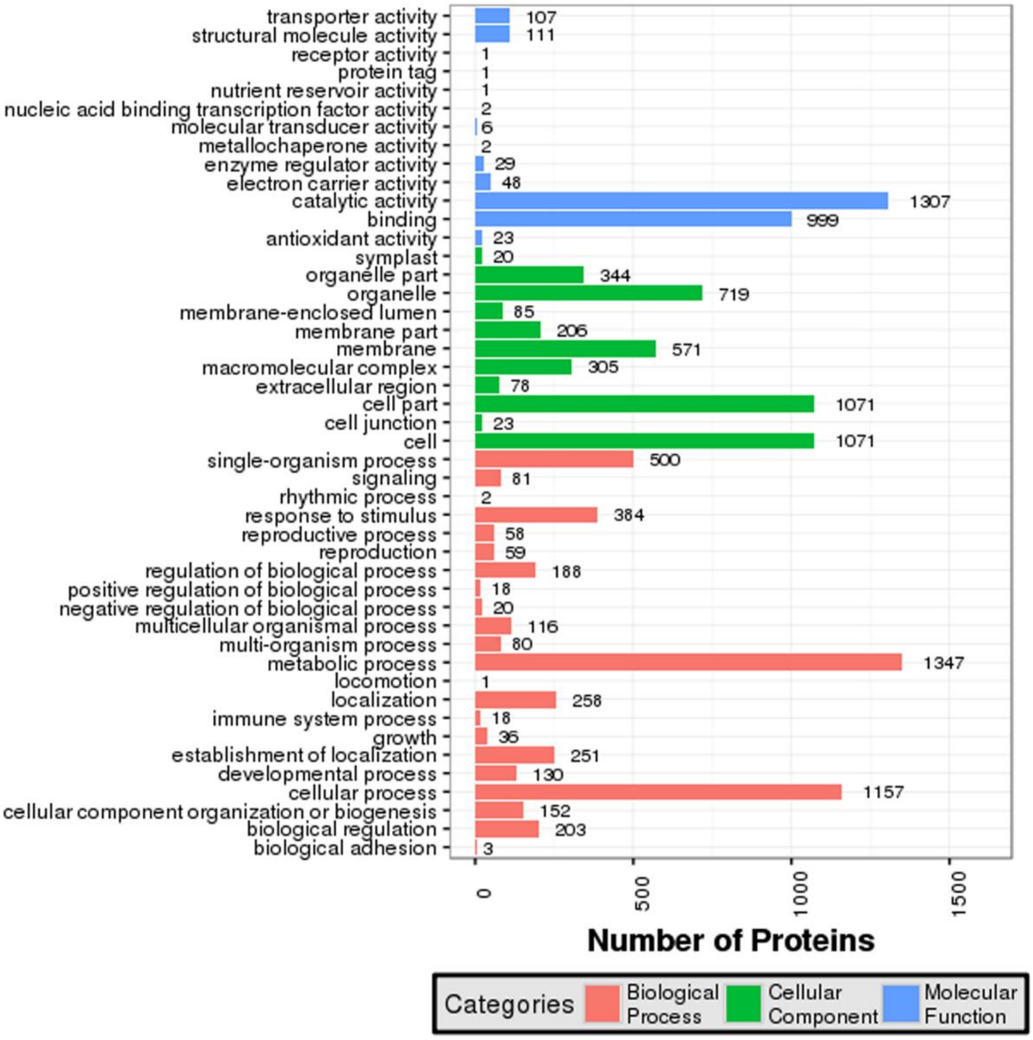

Figure S5. Cluster analysis of correlated DEGs and DEPs at different developmental time points. Each row in the figure represents a DEP/DEG, with its ID and differential expression multiple on the right. Red represents up-regulated expression and green represents down-regulated expression.

32 DAP-vs- 25 DAP

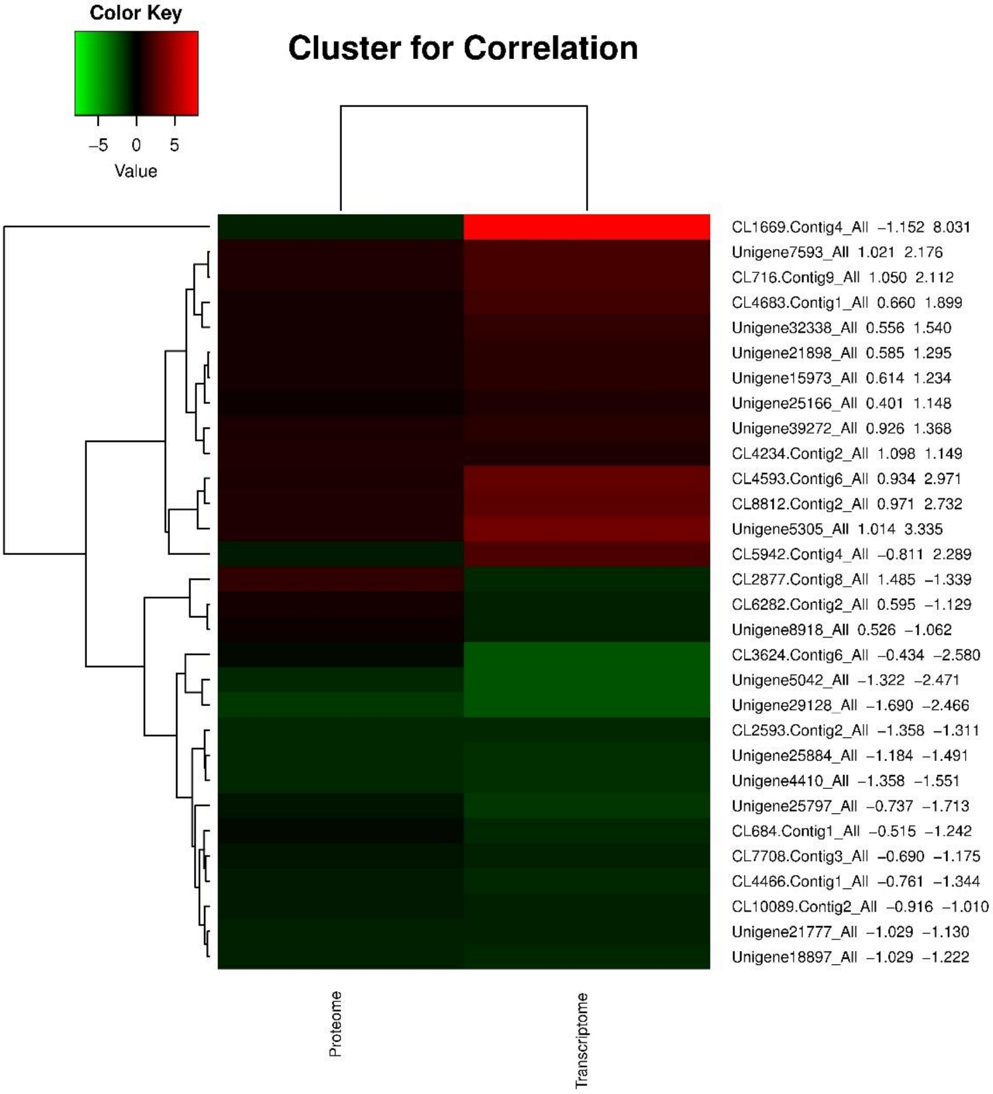

39 DAP-vs- 25 DAP

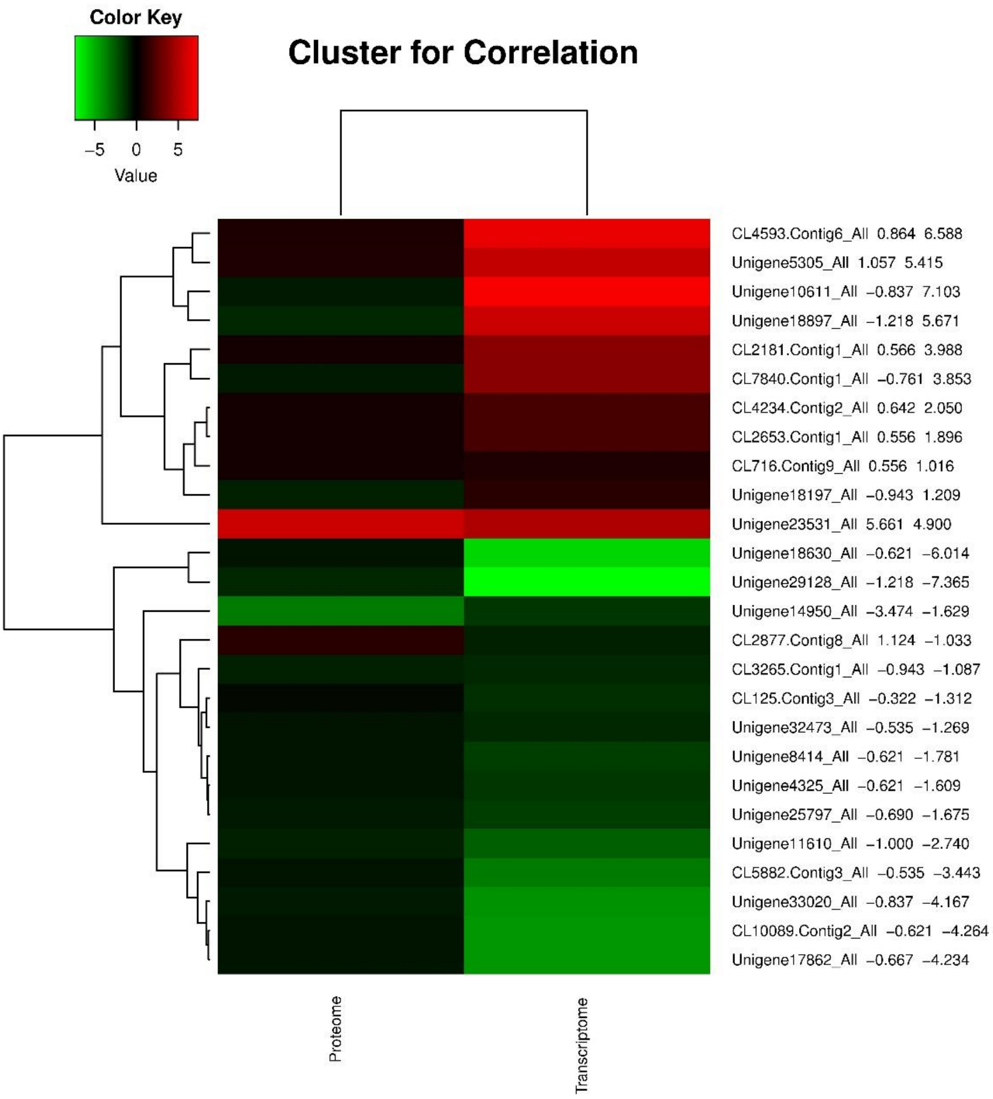

53 DAP-vs- 25 DAP

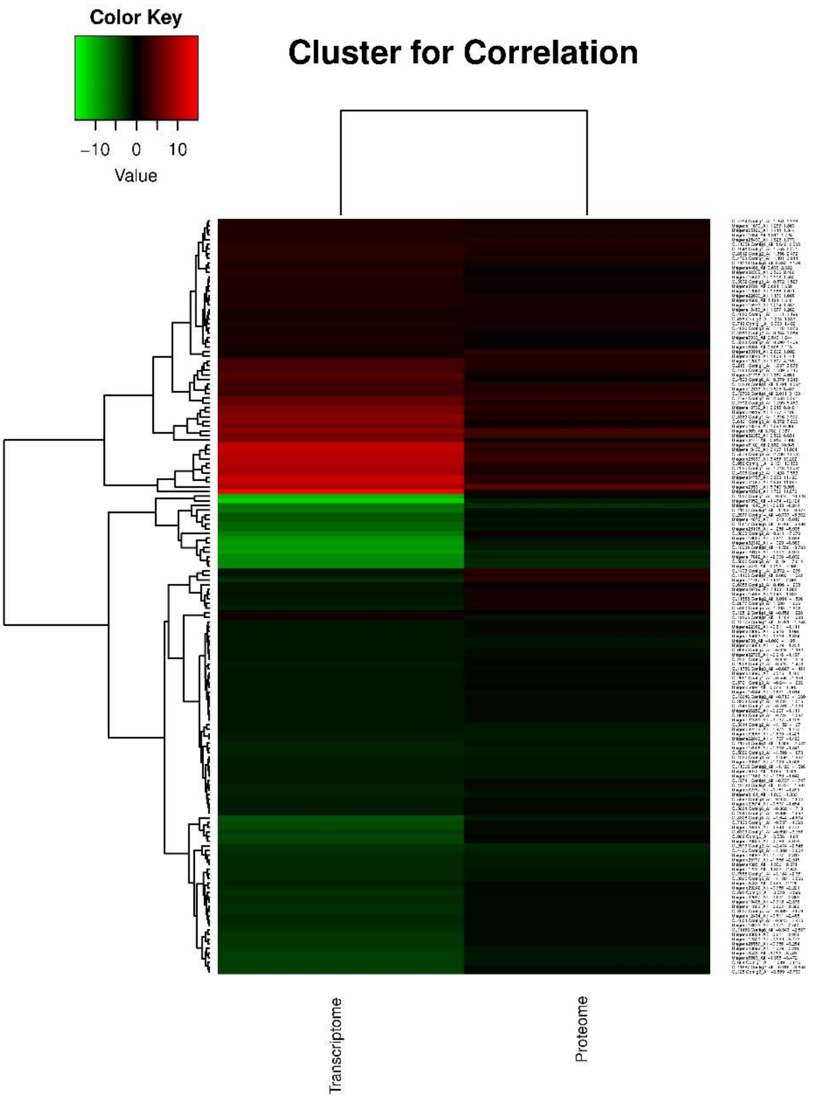

67 DAP-vs- 25 DAP

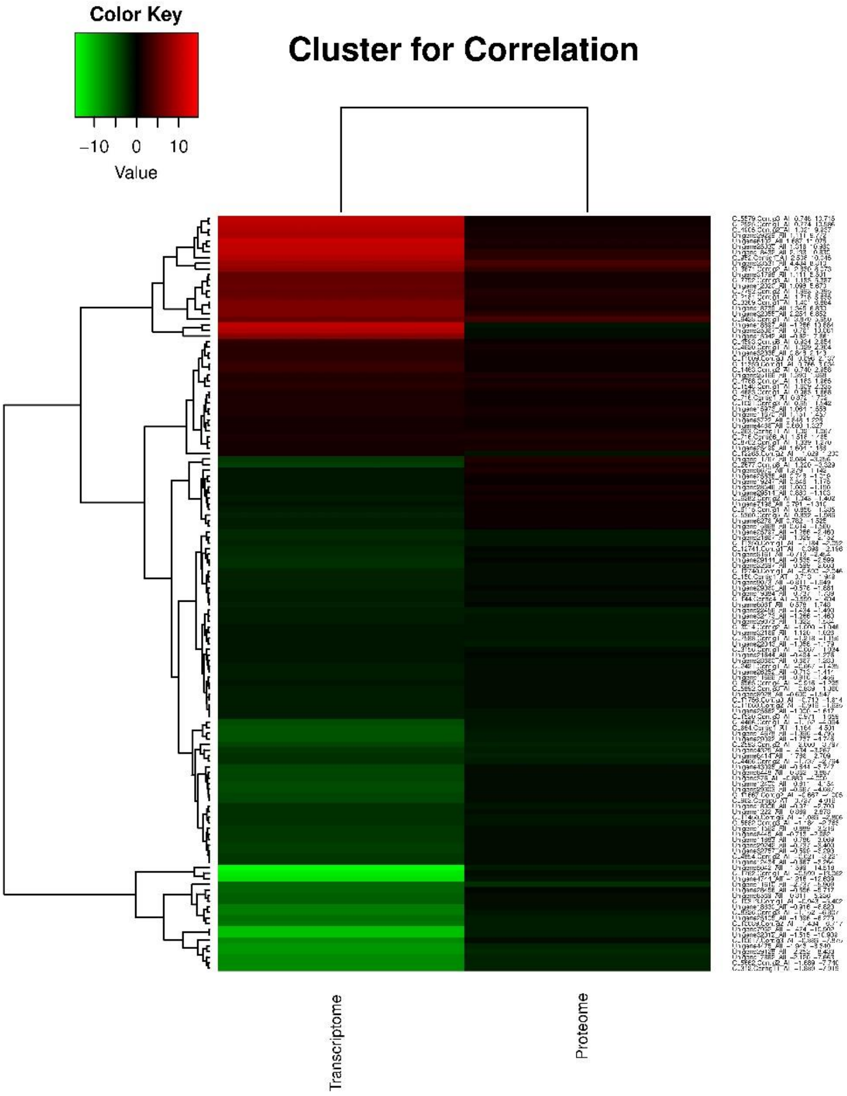

81 DAP-vs- 25 DAP

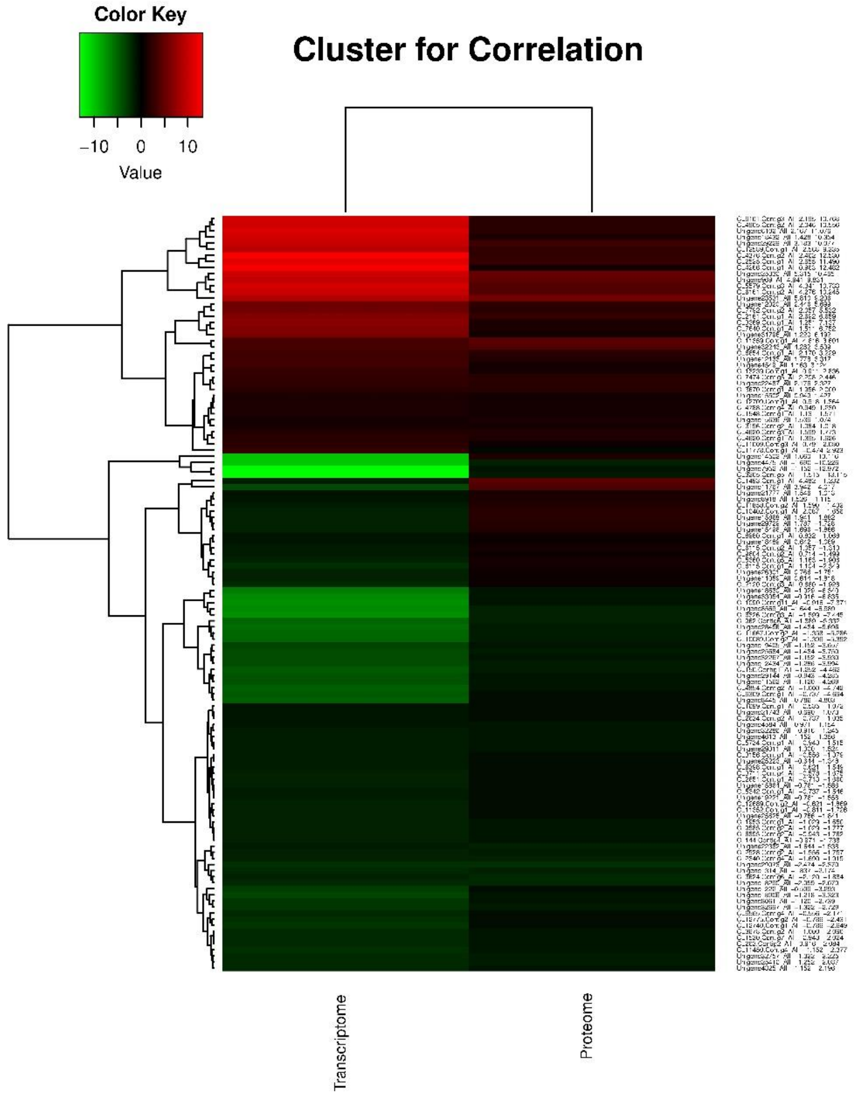

88 DAP-vs- 25 DAP

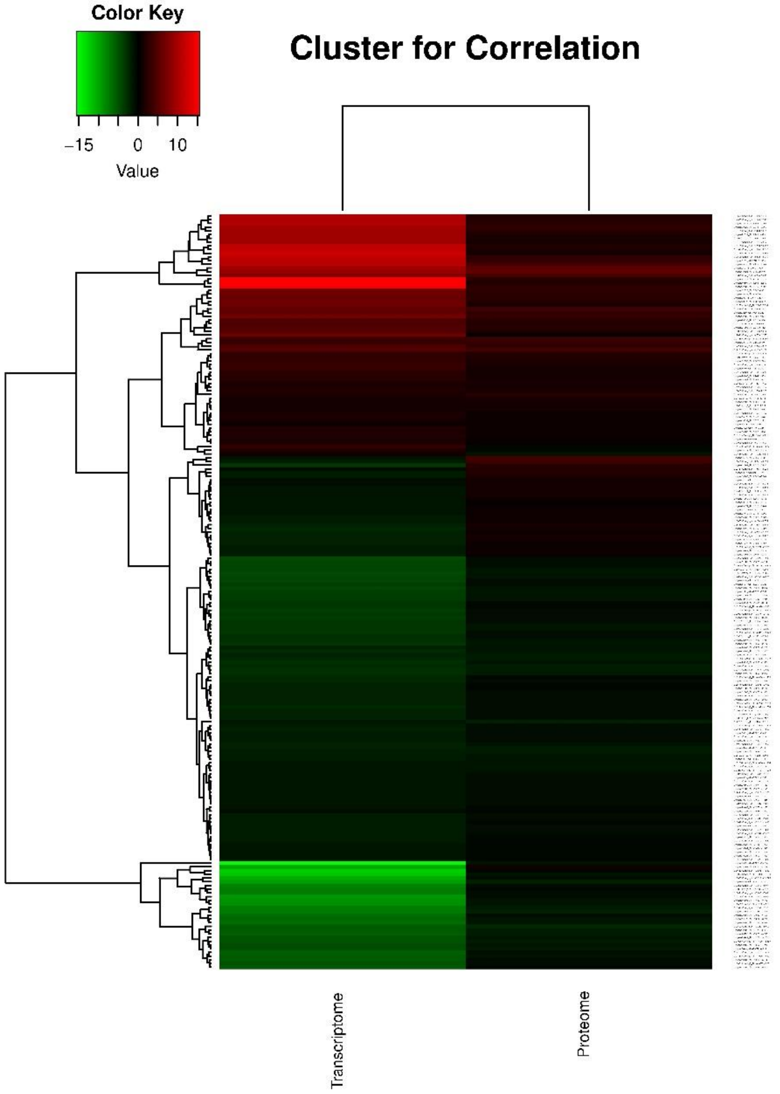

95 DAP-vs- 25 DAP

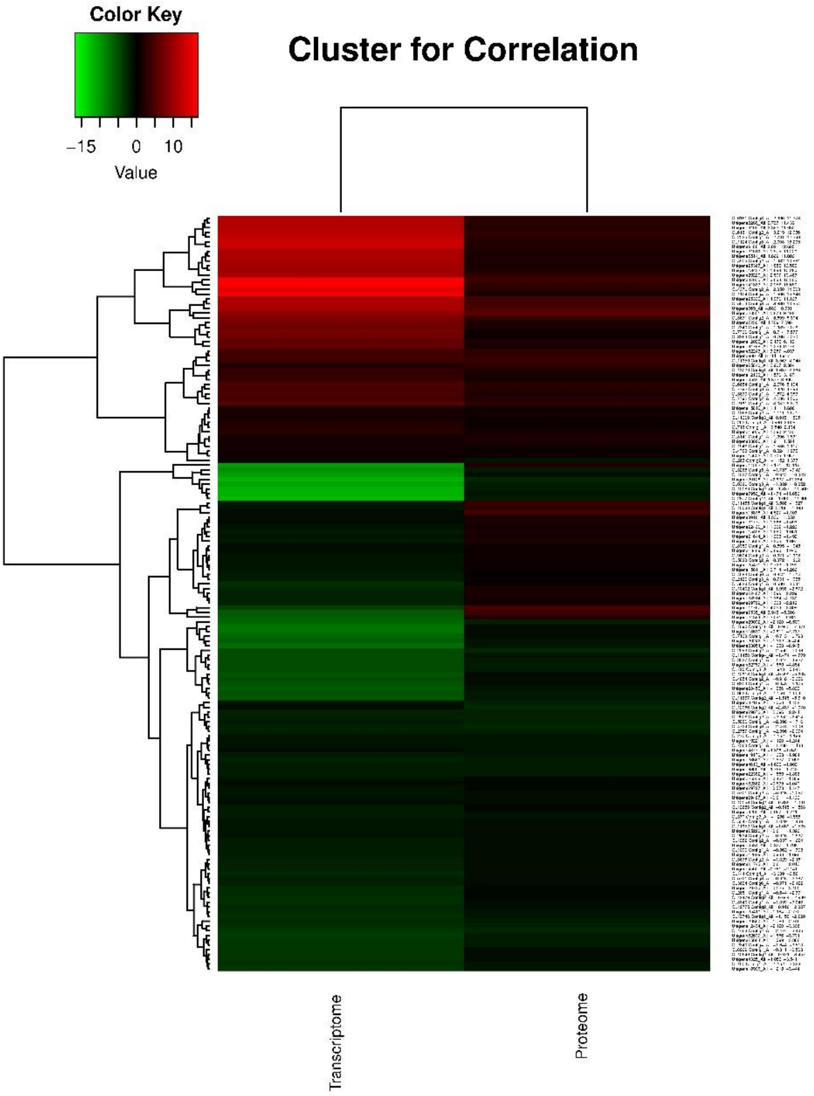

109 DAP-vs- 25 DAP

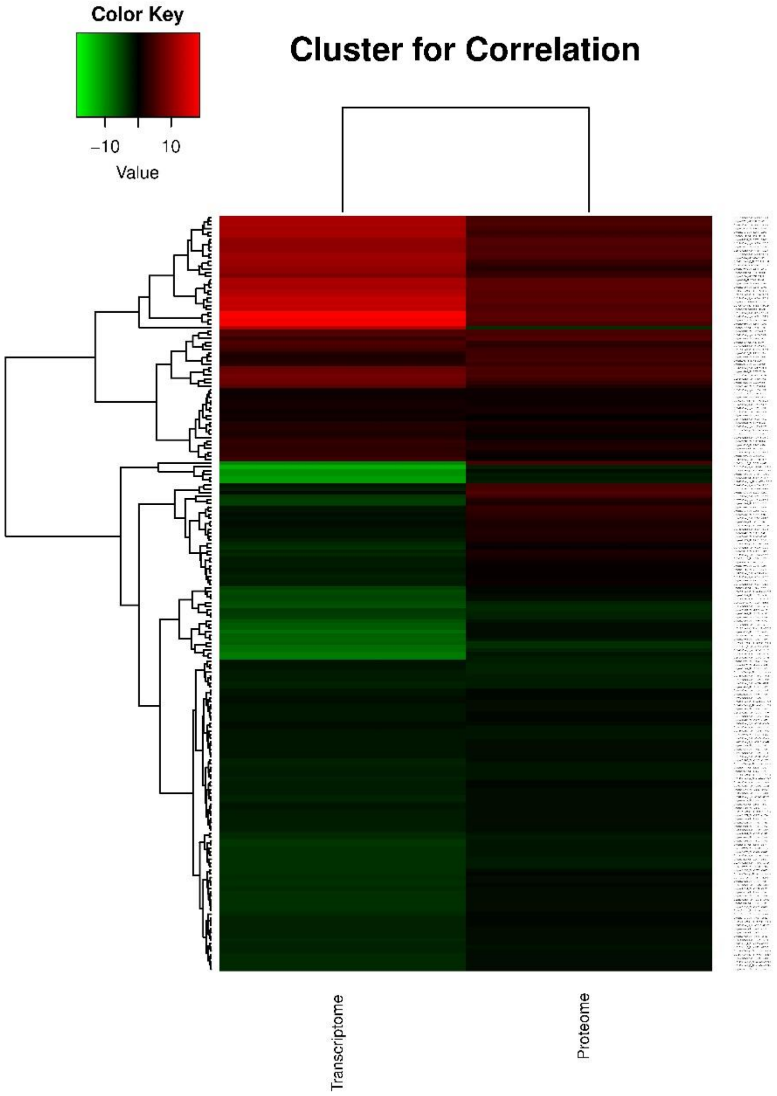

Supplement: Supplementary file 1 — Supplementary Table and Supplementary Figure. [file 41438_2019_194_MOESM1_ESM.pdf]
